# Specificity of Episodic Future Thinking in Adolescents: Comparing Childhood Maltreatment, Autism Spectrum, and Typical Development

**DOI:** 10.1007/s10802-024-01232-7

**Published:** 2024-08-21

**Authors:** A. Lau-Zhu, C. Chan, D. Gibson, E. Stark, J. Wang, F. Happé, J. Stacey, M. Cooper

**Affiliations:** 1 Department of Experimental Psychology, Medical Sciences Division, University of Oxford, Oxford, UK; 2 Division of Psychiatry, Department of Brain Sciences, Imperial College London, London, UK; 3 Child and Adolescent Mental Health Services, Oxford Health NHS Foundation Trust, Oxford, UK; 4 Department of Psychiatry, Medical Sciences Division, University of Oxford, Oxford, UK; 5 Centre for Eudaimonia and Human Flourishing, Linacre College, University of Oxford, Oxford, UK; 6 Child and Adolescent Mental Health Services, Berkshire Healthcare NHS Foundation Trust, Reading, UK; 7 Social, Genetic, & Developmental Psychiatry Centre, Institute of Psychiatry, Psychology and Neuroscience, King’s College London, London, UK

**Keywords:** Maltreatment, Trauma, Autism, Future Thinking, Episodic Simulation, Mental Imagery

## Abstract

Maltreatment and autism can be associated with overlapping difficulties across functional domains (e.g., social, emotional, and sensory) and high rates of mental health problems. A cognitive approach focussing on affect-laden cognition, here on episodic future thinking (FT), could help inform cognitive assessments and adapt psychological interventions. Three groups of adolescents (*N* = 85), (i) maltreatment (*n* = 28), (ii) autism (*n* = 29), and (iii) typical development without maltreatment/autism (TD; *n* = 28), matched in age (10–16 years old), sex (assigned at birth), and socioeconomic status, completed a newly adapted online Autobiographical Future Thinking Test. As predicted, the maltreatment group generated significantly fewer specific future events relative to the TD group, however, the number of specific future events did not significantly differ between the autism and the other groups. Exploratory analyses showed that lower FT specificity was significantly associated with more depressive (but not anxiety) symptoms across the three groups. These findings shed light on the cognitive profiles of both maltreatment and autism during adolescence and signal FT as a potential therapeutic target for adolescents with these developmental differences. Our study lays the foundation for additional comparisons of maltreatment-related presentations versus autism with improved designs and a broader set of cognitive and clinical domains.

**Supplementary Information:**

The online version contains supplementary material available at 10.1007/s10802-024-01232-7.

## Introduction

### Maltreatment or Autism?

“Is it because of developmental trauma or autism^1^?” Disentangling whether a child’s presenting difficulties result from either (or both) is an enquiry gaining scientific and clinical traction (Davidson et al., 2022; Minnis et al., 2020; Moran, 2010). Overlapping difficulties have been reported between maltreatment-exposed^2^ and autistic young people (YP) across functional domains such as social interactions, emotional regulation, and sensory processing (Davidson et al., 2015; Rutter et al., 2007). Such overlap can complicate differentiation for practitioners (Moran, 2010), which is important for tailoring trauma- and neurodiversity-informed support. This dilemma is further compounded by the high rates of co-occurring mental-health problems (Gilbert et al., 2009; Lai et al., 2019). It remains paramount to develop more effective interventions for emotional psychopathology in these populations (e.g., Lau-Zhu & Vella, 2023), given limited gains observed for them from gold-standard psychological interventions, including cognitive-behavioural therapies (Lippard & Nemeroff, 2020; Weston et al., 2016).

Childhood maltreatment refers to abuse and neglect typically from someone in a position of power or responsibility (May-Chahal & Cawson, 2005). Autism spectrum disorder is a neurodevelopmental condition with a strong genetic basis (Tick et al., 2016), characterised by difficulties/differences with social communication/interaction, need for sameness, and sensory sensitivities (APA; American Psychiatric Association, 2013). There is a high co-occurrence of autism and maltreatment (Dinkler et al., 2017; Hoover, 2015; McDonnell et al., 2019), likely due to shared familial vulnerability including genetic factors (Danese et al., 2017; Dinkler et al., 2017). Nevertheless, such “double jeopardy” could mutually reinforce each other over time, producing additive effects on severe and enduring mental health outcomes (Gajwani et al., 2021). A proposed mechanism for these involves stress systems that underpin emotional regulation (Gajwani & Minnis, 2023).

### A Cognitive Approach

Cognitive science offers tools to examine mental processes, thus to understand developmental differences (Frith, 2001) and improve mental health (Harvey et al., 2004). Substantial parallel literatures have revealed cognitive differences among maltreatment-exposed (e.g., McCrory & Viding, 2015) or autistic individuals (e.g., Brunsdon & Happé, 2013) relative to TD peers, for example in theory of mind (Benarous et al., 2015; Tager-Flusberg, 2007), emotion recognition (Uljarevic & Hamilton, 2013; Yeung, 2022), and autobiographical memory(Goodman et al., 2010; McDonnell et al., 2017).

A recent developmental psychopathology theory has proposed that childhood maltreatment can result in calibration of the neurocognitive system to manage the associated “toxic stress”, reflected in adaptive yet biased information processing in attention, memory and emotion (McCrory & Viding, 2015). These “latent vulnerabilities” increase risks for later psychopathology in adulthood but are thought to be already observable in childhood. This theoretical account thus underscores the importance of studying cognitive processes in maltreatment and associated presentations.

Direct comparisons between maltreatment and autism have recently emerged but primarily on clinical observations (Moran, 2010) or symptom reports (Coughlan et al., 2021; Minnis et al., 2020), neglecting cognition. One exception is by Davidson et al. (2015), who found that autistic (but not maltreatment-exposed) youth more likely had marked discrepancies between verbal and non-verbal IQ. While these findings could inform differential assessments, they map onto “cold” (emotion-independent) rather than “hot” (emotion-laden) cognition (Roiser & Sahakian, 2013), that is, affect-laden and self-relevant information-processing biases most critical to emotional psychopathology (Harvey et al., 2004).

### Episodic Future Thinking

Episodic future thinking (FT) represents a form of “hot” cognition that involves simulating the future often recruiting mental imagery (Schacter et al., 2017), a representational format with privileged links to emotions (Ji et al., 2019). FT has been theorised to be central for planning, problem solving, identity formation, social bonding, and emotional regulation (Akbari et al., 2023; Szpunar et al., 2014), functional domains often affected in autistic and maltreatment-exposed individuals (McKenzie & Dallos, 2017; Moran, 2010). Empirically, simulation of personal future episodes has been associated with improved action planning (D’Argembeau et al., 2011), modulation in stress and worry (Brown et al., 2002), increased motivation to enact planned behaviours (Ji et al., 2021), better problem-solving (Jing et al., 2019), and accessing of personal knowledge (D’Argembeau & Mathy, 2011).

In typically developing (TD) adults, difficulties in generating specific (i.e., temporally- and spatially-bound) mental representations involving the self in the future have been reported across psychiatric disorders, particularly depression (Gamble et al., 2019; Hallford et al., 2018). Marked development in FT coincides with adolescence when many begin to envision their future selves in terms of education, careers, and relationships (Johnson et al., 2014; Nurmi, 1991). Thinking, anticipating and planning for the future (Steinberg et al., 2009) can play an important role in buffering against the negative effects of childhood adversity on socio-emotional development (Cui et al., 2020), thus FT has clinical relevance for preventative and early interventions.

Our focus on FT is partly inspired by our interactions with families (e.g., in clinical services), who have reported that talking about the future in particular can be difficult for their young people who were exposed to maltreatment. Unlike other neurocognitive domains such as attention and memory, FT has not yet been examined as a potential “latent vulnerability” following maltreatment (McCrory & Viding, 2015). Any FT-related atypicalities, however, could also be explained by co-occurring autism. Autistic individuals’ struggles with insistence on sameness and behavioural inflexibility may partly be because of challenges with pre-experiencing future events, limiting their ability to plan optimally and flexibly (Lind & Bowler, 2010). Investigations of FT in maltreatment thus ought to also consider autism, given its potential significance in both developmental presentations and the high maltreatment-autism co-occurrence, as explained previously. Findings on a youth sample also holds relevance for investigating FT’s potential as maintenance as well as developmental risk factor for psychopathology.

Predictions on episodic FT can be derived from research on episodic memory given a shared neurocognitive system (Schacter & Addis, 2007). Childhood maltreatment has been linked to reduced memory specificity in YP and adults (Kuyken et al., 2006; McCrory et al., 2017; Valentino et al., 2009), with recent studies also suggesting that FT is less specific in trauma-exposed adults (Brown et al., 2013; Kleim et al., 2014; Maccallum & Bryant, 2011; Robinaugh & McNally, 2013). In trauma-exposed individuals, reduced memory (and FT) specificity could facilitate affect regulation, as painful memory details can be avoided, but over time resulting in a habitual cognitive style applied indiscriminately to all memory content (Hitchcock et al., 2017). Direct evidence is however lacking in trauma-exposed YP for levels of FT specificity (including the role of avoidance).

Similarly for autism, autobiographical memories have been shown to be less specific for YP/adults relative to TD peers (McDonnell et al., 2017), with findings extending to FT in adults (Feller et al., 2021; Lind & Bowler, 2010). For autistic youth, FT specificity has yet to be studied, although FT seems to be characterised by a reduced number of *details* (Ciaramelli et al., 2018; Lind et al., 2014; Terrett et al., 2013). This pattern of findings may seem surprising given evidence regarding a detail-focussed processing style in autism (Happé & Frith, 2006). Reduced memory and FT specificity in autism could be driven by reduced executive functioning– a core challenge in autism(O’Hearn et al., 2008)– likely impairing one’s ability to (re)construct a specific event in mind by joining multiple sensory details (McDonnell et al., 2017). Overall, it remains unexplored whether episodic FT specificity is reduced in maltreatment-exposed and autistic youth: is this a cognitive similarity or difference?

### The Present Study and Hypotheses

In a cross-sectional study, we provide a first direct comparison between three groups of adolescents (aged 10–16 years) with: (i) a diagnosis of ASD, (ii) a history of maltreatment, and (iii) typical development without ASD/maltreatment (TD for simplicity), using a novel online test of FT specificity adapted from a well-established autobiographical memory paradigm (Heron et al., 2012; Williams et al., 1996). We focused on understanding the profiles of “pure” presentations first (i.e., ASD only or maltreatment only) as these could help illuminate cases where there may be presentation ambiguity in future.

Our primary aim was to examine levels of specificity using an online test to facilitate further work in this area where in-person recruitment of children and families can be challenging. Based on the reviewed literature, we hypothesised that both maltreatment-exposed and autistic adolescents will generate significantly fewer specific future events than TD adolescents. Ascertaining difficulties in FT within each of these developmental groups is important in their own right to inform assessments and interventions.

Our secondary aims were to explore cognitive/clinical correlates of FT specificity in adolescents. This enabled us to investigate if the groups differed in the potential underlying mechanisms driving any primary effects. We hypothesised significant associations between (i) FT specificity and avoidant coping (especially within the maltreated-exposed group), and (ii) FT specificity and executive functioning (especially with the autistic group). We also predicted significant links between reduced FT specificity and mental health (anxiety and depression) across groups.

## Method

### Participants

Eighty-five adolescents aged 10–16 years (and a caregiver) took part. A small donation was given to a youth charity for each adolescent. Based on a large effect (Cohen *d* = 0.79, alpha = 0.05, power = 0.80, and two-tailed tests) from a prior study on memory specificity in autistic YP (Goddard et al., 2014), and moderate-to-large effects (i.e., Cohen *d* = 0.50–0.80) from a review on memory specificity in trauma-exposed YP (Hitchcock et al., 2014), 27 adolescents per group was needed to detect significant group differences between autism/maltreatment and TD.

The autism group (*n* = 29) was recruited via local mental health services and community advertisements such as through charities. All adolescents had a diagnosis of ASD based on established criteria in the Diagnostic and Statistical Manual (DSM) 4th (APA, 1994) or 5th editions (APA; 2013), or the International Classification of Diseases (ICD) 10th (WHO; World Health Organization, 2016) or 11th editions (WHO; 2019), as confirmed by keyworkers (who had access to clinical records) or caregivers (who provided written evidence, if needed). Keyworkers/caregivers also confirmed no maltreatment history nor prior social service contact.

The maltreatment group (*n* = 28) was recruited via local social and mental health services with documented maltreatment history, confirmed by keyworkers (who had access to health/social care records). Maltreatment was defined as sexual abuse, physical abuse, emotional abuse and/or neglect, informed by established descriptors (Kaufman et al., 1994). Keyworkers established the absence of an ASD diagnosis or of concerns about the child potentially being autistic (e.g., diagnosis being considered). Caregivers confirmed the absence of first-/second-degree relatives with ASD.

The TD group (*n* = 28) was recruited via community advertisements (e.g., social media and university-wide advertisement) and word of mouth (e.g., asking families to share information). Caregivers confirmed that adolescents did not have: an ASD diagnosis (nor were being considered for one); first/second-degree relatives with an ASD diagnosis; a history of abuse/neglect; or prior social service contact (due to the child’s quality of care). As TD participants were not recruited via statutory services they were not linked to keyworkers.

Exclusion criteria for all adolescents (based on caregiver-reports) were: (1) not reading English; (2) an organic brain condition (e.g., brain injury); (3) a history of psychotic episodes; (4) a diagnosis of learning disability (or known IQ < 70); 4) actively suicidal; (5) severe sensory impediments that would markedly disrupt task completion (e.g., difficulties with screen exposure > 15 min). We did not attempt to exclude comorbidities at the recruitment stage (e.g., anxiety/depression diagnoses) or match all three groups on these given the now widespread recognition that mental health problems are more often the norm than the exception in autistic (Gillberg & Billstedt, 2000; Happé & Frith, 2020; Lai et al., 2019) and maltreatment-exposed individuals (Ford et al., 2007; Meltzer et al., 2003).

Online consent was obtained from adolescents aged 16 and from the caregiver or legal guardian for adolescents aged below 16, who also provided online assent. Ethics approval was obtained from West Midlands - Solihull Research Ethics Committee (21/WM/0125).

### Measures

The whole study was conducted online using Qualtrics software (Qualtrics, 2020). Separate surveys were created for adolescents and caregivers. They were instructed to be in a quiet environment without distractions before proceeding with the survey. Median completion time was 25 min for adolescents and 15 min for caregivers.

#### Autobiographical Future Thinking Test

The Autobiographical Future Thinking Test was adapted from the Autobiographical Memory Test (AMT) used in adolescents (Kuyken et al., 2006; Valentino et al., 2009; Vrielynck et al., 2007), which was in turn adapted from the original version developed for adults (Williams et al., 1996). Previous work has successfully adapted the AMT as a written questionnaire and found evidence of a single continuous trait (Heron et al., 2012; Takano et al., 2017).

Ten word cues (positive: happy, safe, interested, successful, surprised; negative: sorry, angry, clumsy, hurt, lonely) were included and previously used with depressed, autistic, or maltreatment-exposed YP (Goddard et al., 2014; Kuyken et al., 2006; Valentino et al., 2009). Instructions were adapted to (i) probe for “future events”, (ii) use simpler language, and (iii) be completed in a written format (Heron et al., 2012). The instructions and format were reviewed by autistic and maltreatment-exposed adolescents, their caregivers, and practitioners, for understanding and feasibility. Adolescents were asked to describe “an event that could happen to you in the future” such as “tomorrow, in a few days or weeks from now, or even when you are much older”. They were also told not think of an event from the past, and given an example of a specific event (e.g. “I will give an important presentation to my class in front of my classmates next Friday” for the word “important”). Participants were also instructed to type “I can’t think of a future event” if that was the case, to ensure they would at least attempt to generate an event for all word cues. To reduce undue pressure and maintain motivation they were also told that “sometimes it’s hard to think of an event in the future but that’s ok. Not everyone can think of something for every word”. Participants could not skip any items and a written answer was required for each (even if to indicate their inability to think of a future event). A time constraint was not imposed.

Future events were coded as *specific* if the event generated would occur on a particular day, time, and place, and *overgeneral* if the event would occur over two days or more or referred to a category of events. Specificity scores were the total number of specific events generated. Coding was performed by the first author with participants’ responses blinded to group membership. A subset of the responses (20%) was double-coded (by another rater also blinded to the group membership) with strong agreement, Cohen’s kappa = 0.83 (Altman, 1991).

#### Questionnaires

See *Supplemental Materials* for more detailed descriptions.

##### For Adolescents

The Revised Children’s Anxiety and Depression Scale– 11 items (RCADS-11; Radez et al., 2021) was used to measure symptoms of anxiety and depression. The Children’s Revised Impact of Event Scale (CRIES; Perrin et al., 2005) was used to measure PTSD symptoms yielding separate subscales for intrusion and avoidance symptoms (the latter serving as an index of avoidant coping). The Abbreviated 9-item form of the Raven’s Standard Progressive Matrices Test (RSPMT-9; Bilker et al., 2012) was used as a proxy for general cognitive ability (GCA; Pind et al., 2003). It is highly predictive of performance on the original 60-item form (Raven, 2000) and has recently been used with adolescents (e.g., Bone et al., 2021; Morin et al., 2019).

##### For Caregivers

The RCADS-47 was used to measure adolescents’ anxiety and depressive symptoms (Chorpita et al., 2005). The Child and Adolescent Trauma Screen (Sachser et al., 2017) was used to measure adolescents’ number of previous traumatic events and PTSD symptoms based on DSM-5 criteria (APA; 2013). The Social Communication Questionnaire– Current version (SCQ; Rutter et al., 2003) was used to measure adolescents’ autistic traits. The Dysexecutive Questionnaire– Children (DEX-C; Emslie et al., 2003) was used to measure adolescents’ executive functioning skills in everyday contexts. Caregivers also provided information on adolescents’ demographic details, known diagnoses, and parental/caregiver highest educational level as a proxy for socioeconomic status (SES; Liberatos et al., 1988).

### Statistical Analyses

Normality was inspected visually with histograms and Shapiro-Wilk tests. For normal continuous variables, overall group differences were assessed with one-way ANOVAs and follow-up independent-sample t-tests where indicated. Homogeneity of variance was assessed using the Levene’s statistic. For non-normal data, including the primary outcome (specificity scores), the overall group difference was assessed with Kruskal-Wallis tests and Mann-Whitney tests for pairwise contrasts where indicated. For the latter, effect sizes were calculated using *r* = z/sqrt(N): 0.10 as small, 0.30 as medium and 0.50 as large (Fritz et al., 2012). For categorical variables, group differences were assessed with Chi-square tests. A two-tailed alpha level of 0.05 was used. Associations between specificity scores and other variables were assessed with Spearman’s rank correlation tests. To minimise the number of correlations performed, principal component analyses (varimax rotation and eigen value > 1) were used to extract a common “depression” and “anxiety” components combining the adolescent and caregiver versions of the RCADS. Correlations were compared with Fisher’s tests. All analyses were conducted in SPSS version 27 (IBM, 2020).

## Results

### Demographic and Clinical Characteristics

Descriptive statistics on key background variables are shown in Table 1 (including information on missing data). The groups did not significantly differ in (chronological) age, sex (assigned at birth), and SES. Based on parental reports, a higher proportion of adolescents described ethnicity as White in the autism group. The maltreatment group scored lowest on the Raven’s measure of GCA.

The autism group reported the highest number of autistic traits, and the maltreatment group reported the highest number of previous traumas. Relative to the TD group, the autism group also reported more previous traumas and the maltreatment group also reported more autistic traits, consistent with the high “overlap” (Gajwani & Minnis, 2023). In the maltreatment group, there were histories of sexual abuse (*n* = 6), physical abuse (*n* = 9), emotional abuse (*n* = 15) and neglect (*n* = 23), with many experiencing more than one form of maltreatment (*n* = 19).

**Table 1:** Background variables including demographics and clinical measures, by groups

	Autism (*n* = 29)	Maltreatment (*n* = 28)	TD (*n* = 28)	Group comparisons^d^	Effect sizes
Age, years: mean (*SD*)	12.52 (1.99)	13.57 (2.08)	12.89 (2.01)	0.146	η_p_^2^ = 0.05
Sex (assigned at birth)^e^: n females (%)	13 (45%)	17 (61%)	14 (50%)	0.474	V = 0.13
Ethnicity: n White (%)	27 (93%)^a^	19 (68%)^b^	17 (61%)^b^	0.013	V = 0.32
Asian, n	0	1	5		
Black, n	1	4	0		
Mixed, n	1	2	3		
Other, n	0	1	3		
Prefer not to say, n	0	1	0		
Diagnoses of neurodevelopmental conditions (others)^f^: *n* (%)	13 (45%)^a^	2 (7%)^b^	0^b^	< 0.001	V = 0.52
Diagnoses of emotional disorders^g^: *n* (%)	8 (28%)^a^	2 (7%)^b^	0^b^	0.004	V = 0.37
Medication, yes: *n* (%)	13 (45%)^a^	1 (4%)^b^	0^b^	< 0.001	V = 0.55
Talking therapy, yes: *n* (%)	13 (45%)^a^	18 (64%)^a^	0^b^	< 0.001	V = 0.56
Socioeconomic status (SES), parental or caregiver education: *n* (%) at university level	20 (69%)	13 (54%)	18 (67%)	0.498	V = 0.13
General cognitive ability (GCA), RSPMT-9^h^: mean (*SD*)	4.85 (2.16)^a^	3.37 (1.50)^b^	5.29 (2.05)^a^	< 0.001	η_p_^2^ = 0.16
Executive functioning, DEX-C: mean (*SD*)	39.72 (15.08)^a^	33.81 (17.49)^a^	8.83 (7.50) ^b^	< 0.001	η_p_^2^ = 0.53
AS traits, SCQ^h^: mean (*SD*); *n* > clinical cut-off	18.03 (5.77)^a^; 20 (69%)	10.77 (4.74)^b^; 7 (27%)	4.75 (3.24)^c^; 0	< 0.001	η_p_^2^ = 0.59
Trauma history; lifetime n of *DSM*-5 traumatic events (CATS^h, i^): *mdn (range)*	1 (0–4)^a^	3 (0–8)^b^	0 (0–4)^c^	< 0.001	*ε*^2^ = 0.34
PTSD symptoms (caregiver report), CATS^i^: mean (*SD*); *n* (%) > clinical cut-off	21.00 (12.21)^a^; 7 (24%)	26.60 (18.10)^a^; 11 (41%)	6.00 (5.33)^b^; 0	0.020	η_p_^2^ = 0.19
PTSD symptoms (self report), CRIES^h^ total score: mean (*SD*); *n (%)* > clinical cut-off	19.21 (12.18)^a^; 16 (55%)	19.07 (13.44)^a^; 15 (54%)	12.46 (10.43)^b^; 9 (36%)	0.063	η_p_^2^ = 0.07
Anxiety symptoms (caregiver report), RCADS-47 *t*-scores: mean (*SD*); *n* (%) > clinical cut-off	74.34 (11.99)^a^; 16 (55%)	67.00 (15.84)^a^; 8 (29%)	49.03 (9.55)^b^; 1 (4%)	< 0.001	η_p_^2^ = 0.34
Anxiety symptoms (self report, RCADS-11 total score: mean (*SD*); *n* (%) > clinical cut-off	7.66 (4.86)^a^; 16 (55%)	7.00 (5.18)^a^; 13 (46%)	4.64 (2.79)^b^; 10 (36%)	0.030	η_p_^2^ = 0.08
Depression symptoms (caregiver report), RCADS- 47 *t*-scores: mean (*SD*); *n* (%) > clinical cut-off	77.28 (18.14)^a^; 17 (59%)	68.39 (15.28)^b^; 12 (43%)	47.44 (6.97)^c^; 0	< 0.001	η_p_^2^ = 0.43
Depression symptoms (self report), RCADS-11 total score: mean (*SD*); *n* (%) > clinical cut-off	5.69 (4.09)^a^; 6 (21%)	6.00 (4.24)^a^; 8 (29%)	3.75 (2.32)^b^; 1 (4%)	0.050	η_p_^2^ = 0.07

Note. TD; typical development; RSPMT-9 = Raven’s Standard Progressive Matrices Test 9-Items Short Form; DEX-C = Dysexecutive Questionnaire Child Version; SCQ = Social Communication Questionnaire Current Version; PTSD = Post-traumatic Stress Disorder; CATS = Child and Adolescent Trauma Screen; CRIES = Child Revised Impact of Events Scale; RCADS = Revised Child Anxiety and Depression Scale

^a, b, c^ Groups with different subscript letters show significant differences

^d^*p*-values for overall group comparisons across the three groups (with effect sizes as follow: η_p_^2^ for one-way ANOVAs, Cramer’s V for Chi-square tests, and *ε*^2^ for Kruskal Wallis test)

^e^ One participant in the autism group (female at birth) identified as transgender

^f^ Diagnoses of neurodevelopmental disorders included ADHD, Tourette’s syndrome, dyspraxia, dyscalculia, dyslexia, and sensory processing disorder

^g^ Diagnoses of emotional disorders included post-traumatic stress disorder, obsessive compulsive disorder, body dysmorphic disorder, depressive disorder, generalised anxiety disorder

Table 1 also presents group comparisons on key clinical characteristics. Caregivers in the autism group reported more formal diagnoses of emotional (e.g., anxiety disorders and major depressive disorder) and other neurodevelopmental conditions (e.g., attention-deficit hyperactivity disorder or dyslexia) in their adolescents relative to caregivers in the other groups. For anxiety and PTSD, both the autism and maltreatment groups had more symptoms relative to the TD group (indexed by both self- and caregiver-reports). For depression, both the autism and maltreatment groups also had more symptoms as indexed by caregiver reports. The autism group self-reported more depressive symptoms than the maltreatment group, which in turn self-reported more depressive symptoms than the TD group.

### Primary Aim: Testing Levels of Future Thinking Specificity

#### Group Comparisons

**Fig. 1:**
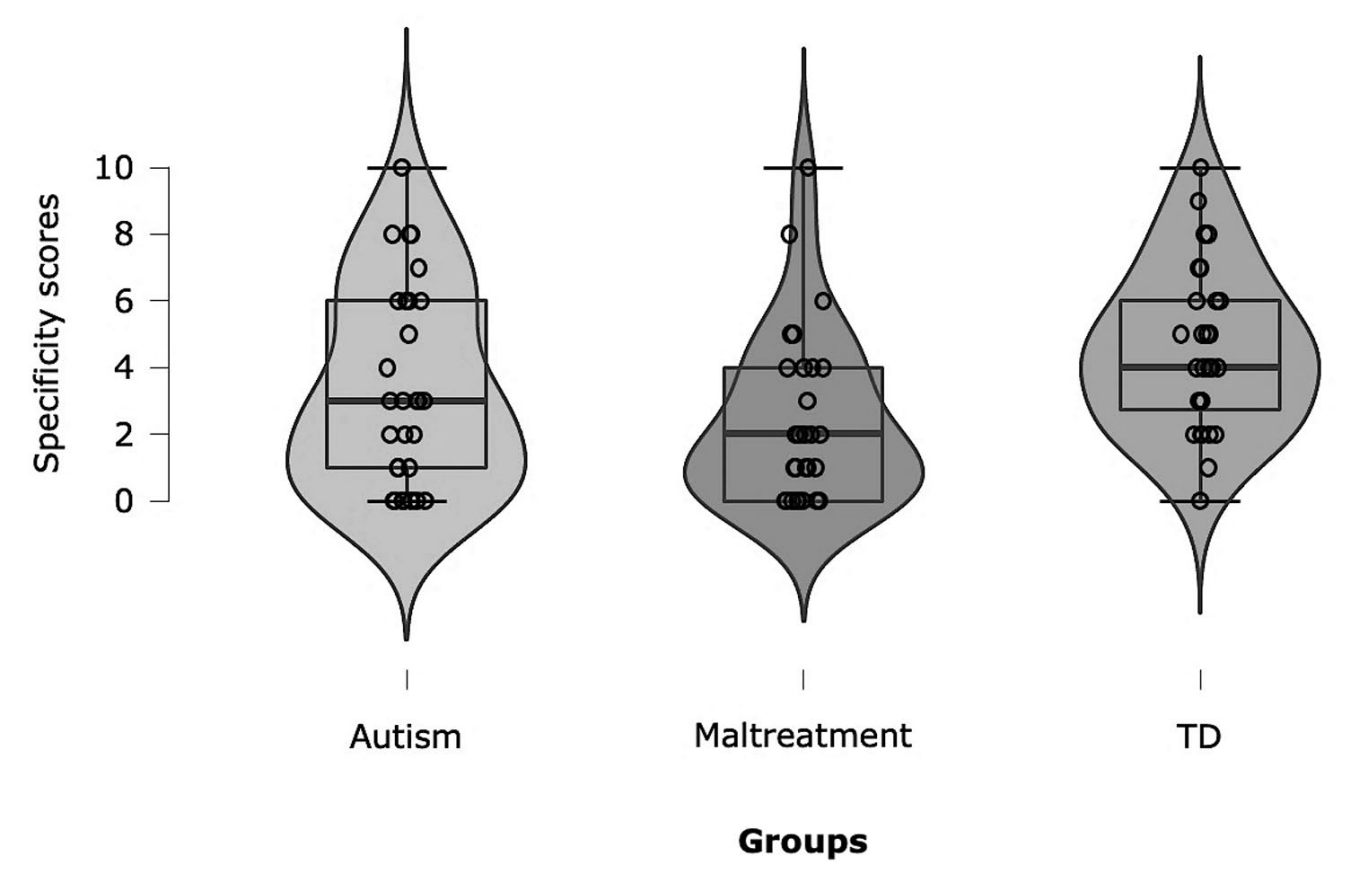
Future Thinking Specificity Scores in Three Groups of Adolescents: Autism, Maltreatment, and Typical Development (TD). Note. Specificity score is the number of specific future events generated in the Autobiographical Future Thinking Test. Each group is represented with jittered raw data, probability density of the data, and boxplot with median and interquartile range

There was a significant overall group difference, *H* = 9.52, *df* = 2, *p* =.009. Planned contrasts revealed that as predicted, the maltreatment group (*Mdn* = 2.00, *IQR* = 0–4.00) generated significantly fewer specific events relative to the TD group (*Mdn* = 4.00, *IQR* = 2.25-6.00), *U* = 199.50, *z* = 3.18, *p* =.001, *r* =.42. However, there were no significant differences between the autism (*Mdn* = 3.00, *IQR* = 0.50-6.00) and TD groups, *U* = 309.00, *z* = 1.69, *p* =.092, *r* =.22, nor between the maltreatment and autism groups, *U* = 333.50, *z* = 1.17, *p* =.241, *r* =.15. The FT specificity data (medians and range) are presented in Fig. 1. Examples of events generated are presented in Table 2.

**Table 2:** Example of events generated in the autobiographical future thinking test

	Examples
Specific	*Getting a distinction in my ballet exam* *Me tripping and scraping my knee* *I’m going to a party*
Extended	*I will feel interested in what I am learning in school over the next few years* *Having friends who will care about me* *Earning lots of money*
Categorical	*Doing something wrong* *Achieving something* *I will be angry if I miss future opportunities due to exams*

No significant correlations were found between FT specificity and age, sex (assigned at birth), and ethnicity, *r*(83)’s = -0.18 to 0.05, *p*’s > 0.094. FT specificity was significantly and positively correlated with SES, *r*(78) = 0.23, *p* =.040, and GCA, *r*(80) = 0.24, *p* =.034. As SES (e.g., Wiley et al., 1998) and GCA (e.g., Heron et al., 2012) have been previously linked to autobiographical memory, we reran the main planned contrasts regressing out SES and GCA. The remaining number of participants per group was as follows: autism (*n* = 27), maltreatment (*n* = 23), and TD (*n* = 27), due to missing data on SES/GCA. FT specificity scores were significantly lower in the maltreatment group compared to the TD group (*U* = 209.00, *Z* = 1.98, *p* =.048). There were no significant differences between the autism and TD groups (*U* = 290.50, *Z* = 1.28, *p* =.200), or the autism and maltreatment groups (*U* = 280.50, *Z* = 0.58, *p* =.559). Therefore, the pattern of significant results remained the same.

**Table 3:** Medians (interquartile ranges) for the number of specific and overgeneral future events by valence

	Autism	Maltreatment	Typical Development
*n*	29	28	28
**Specific**			
Positive	2.00 (0–3.00)	1.00 (0–3.00)	2.50 (1.00–3.00)
Negative	1.00 (0–3.00)	1.00 (0–2.00)	2.00 (1.00–3.00)
**Overgeneral**			
Positive	2.00 (0–2.00)	1.00 (0–2.00)	2.00 (1.00–3.00)
Negative	1.00 (0-2.50)	1.00 (0–2.00)	1.00 (0–2.00)

The same pattern of significant group differences was also found when separating the specificity scores by cue type (positive and negative words; see Supplemental Materials for details). We explored the overall group difference in the number of *overgeneral* (categorical plus extended) events, as this has been a key outcome in some studies (Barry et al., 2021; Sumner et al., 2010), but this was not significant, *H* = 2.67, *df* = 2, *p* =.263. See Table 3 for descriptives of both specific and overgeneral future events (by valence).

#### Sensitivity Analyses

The primary planned comparisons were rerun in separate analyses to explore whether other variables could potentially account for the maltreatment-related findings on FT specificity. To rule out the role of known comorbidities, adolescents declaring other diagnoses (Table 1) were excluded. Given the high numbers of adolescents meeting thresholds in clinical measures, which are not unexpected in a autism/maltreatment sample (Table 1), we excluded adolescents meeting clinical thresholds for overall depression and anxiety (RCADS-47 or RCADS-11) and PTSD (CATS or CRIES) in separate analyses. To test for the possibility of group “misallocation”, we excluded adolescents in the autism group who scored below the cut-off for autism on the SCQ, and adolescents in the maltreatment group who scored above the cut-off. To rule out the possibility of reduced motivation to complete the test, we excluded adolescents who did not generate any specific events. There were also no significant group differences in the number of omissions (e.g., “I can’t think of any events”), indicating that the maltreatment group had not just simply generated fewer future events overall.

Despite these procedures, the different analyses yielded a similar pattern of findings. These therefore increase confidence that we can attribute the findings on FT to maltreatment exposure. Please see *Supplemental Materials* for detailed results for each sensitivity analysis.

### Secondary Aims: Exploring Correlates of Future Thinking Specificity

#### Potential Mechanisms

In the maltreatment group, specificity scores were *positively* and significantly correlated with scores on the CRIES avoidance subscale, *r*(26) = 0.54, *p* =.003, suggesting that more avoidant coping was associated with *more* specificity (rather than with *less* specificity as predicted). To understand this intriguing finding, we explored the same relationship in the other groups. In both the autism and TD groups, specificity scores were *negatively* and significantly correlated with avoidant coping, *r*’s = -0.39 to -0.42, *p*’s < 0.045. The correlation between specificity and avoidant coping was significantly bigger in the maltreatment group compared to the other two groups (Z’s > 3.62, *p*’s < 0.001). Despite the moderate effect size, this correlation in the maltreatment group was no longer significant when using SES- and GCA-regressed specificity scores, *r*(21) = 0.32, *p* =.134 (potentially due to the loss of power with some missing data on SES/GCA). In exploratory analyses, overgenerality scores and avoidant coping were not significantly correlated (see *Supplemental Materials*).

In the autism group, specificity scores (or overgenerality scores; see *Supplemental Materials*) were not significantly correlated with executive functioning (DEX-C), *r*(26) = -0.14, *p* =.485, nor was this the case in the other groups, *r*’s = -0.11 to 0.05, *p*’s > 0.593. The pattern of results remained the same using SES- and GCA-regressed specificity scores.

#### Associations with Mental Health

Across the three groups, less FT specificity was significantly correlated with more symptoms of depression, *r*(85) = -0.23, *p* =.033, but not of anxiety, *r*(85) = -0.17, *p* =.129. When split by group, depression was significantly correlated with specificity scores in the autism group, *r*(29) = -0.39, *p* =.037, but not in the other two groups, *r*(28)’s = -0.04 to 0.11, *p*’s > 0.583. This correlation was significantly bigger in the autism group compared to both the maltreatment group (Z = 1.86, *p* =.031) but not to the TD group (Z = 1.33, *p* =.092). The pattern of results remained the same with SES- and GCA-regressed specificity scores. Repeating these analyses with overgenerality scores did not yield significant results (see *Supplemental Materials*).

## Discussion

FT is salient during adolescence (Johnson et al., 2014; Steinberg et al., 2009) and is proposed to influence socio-emotional development (Cui et al., 2020). We compared maltreatment-exposed, autistic, and TD adolescents in the specificity of self-relevant future events generated. To the best of our knowledge, this is the first study to (i) directly compare a form of “hot” cognition central to emotional disorders (Gamble et al., 2019; Hallford et al., 2018) between maltreatment versus autism groups; (ii) examine FT in maltreatment-exposed YP, exploring whether it could be a potential “latent vulnerability” (McCrory & Viding, 2015); (iii) extend current work on FT in autism (e.g., details) to explore *specificity*; and (iv) adapt an online written test for this purpose (Heron et al., 2012), demonstrating it can sensitively capture individual differences in our intended population. By bridging separate literatures on autobiographical memory (Goodman et al., 2010; McDonnell et al., 2017), we contribute to characterising the multifaceted cognitive profiles associated with maltreatment (McCrory & Viding, 2015) and autism (Brunsdon & Happé, 2013).

For our primary aim, maltreatment-exposed adolescents generated fewer specific future events compared to TD peers (with a medium-to-large effect of *r* =.42) as predicted, whereas there were no significant differences between the autism group and the other groups. The reduced specificity did not simply reflect generating fewer events overall (of any kind), nor it was equivalent to increased overgenerality. These patterns of findings remained after a series of sensitivity analyses (e.g., to account for SES, GCA, comorbid psychopathology and low motivation). It is now widely recognised that mental health difficulties often occur in autistic and maltreatment-exposed individuals, even at subclinical levels. As analyses adjusting for co-occurring anxiety/depression symptoms may inadvertently remove variance linked to autism/maltreatment and reduce generalizability, we instead applied a sensitivity analysis excluding those with symptoms above clinical cut-offs and known comorbid diagnoses. The key patterns of results retained, increasing our confidence that we can attribute the effects to maltreatment exposure. For our secondary aims, lower FT specificity was significantly associated with avoidant coping (with more avoidant coping in the autism/TD groups versus less avoidant coping in the maltreatment group– although not significant when SES and GCA were controlled for) but not with executive functioning. FT specificity was also associated with depression symptoms, particularly in the autism group. Thus, specificity scores were able to sensitively capture individual differences.

We provide a first demonstration that reduced FT specificity extends from trauma-exposed adults (Brown et al., 2013; Kleim et al., 2014; Maccallum & Bryant, 2011; Robinaugh & McNally, 2013) to maltreatment-exposed youth. This finding parallels prior work on reduced *memory* specificity in maltreatment-exposed YP (McCrory et al., 2017; Valentino et al., 2009). Keyworkers corroborated maltreatment status with multi-informant and historical data, which reduces retrospective biases in self-reports (Moore & Zoellner, 2007). Our FT-related findings are related to an objective measure of trauma, even though recent studies highlight the importance of subjective measures (Baldwin et al., 2019), though we note that measurement of trauma is a complex and evolving area (Lacey & Minnis, 2020). As common in cognitive/experimental psychology, our FT test was appropriate for detecting group differences but further psychometric evaluations are needed for using it at an individual level (e.g., clinical assessments). The group-level findings, nevertheless, suggest that FT specificity could represent another “latent vulnerability” (McCrory & Viding, 2015): maltreatment-exposed youth may be “stuck” in the present/past, struggling with the generation of specific future scenarios, in turn reducing motivation for activity engagement, problem solving skills, and ability to remain hopeful. This proposal requires an additional longitudinal follow-up to confirm the predictive value of FT specificity on future psychopathology in a maltreatment sample.

The absence of an autism-related reduction in FT specificity contradicts the memory literature (McDonnell et al., 2017) and recent FT studies in autistic adults (Feller et al., 2021; Lind & Bowler, 2010). As argued by Crane et al. (2013), a written/online test as in our study could have minimised performance disadvantages that are driven by social anxiety, a common concern in autistic individuals (Spain et al., 2018). This finding is against a pervasive notion that autistic people have poor “imagination” (APA, 2013), and instead suggests that autistic adolescents can perform as well as TD adolescents in a task tapping into imagining of future events. Alternatively, FT specificity may be reduced in both developmental presentations but perhaps more profoundly in maltreatment-exposed than autistic adolescents, informing continuous refinement of differential assessments (e.g., Davidson et al., 2015). This could be clarified with larger samples to detect much smaller effects (possibly present in the autism group, *r’s* = 0.15–0.22), while contrasting different test formats, although this possibility requires a sample size of 963 per adolescent group (nearly *N* = 3000 for replicating the whole study). For now it appears that the current online test can most readily capture diminished FT specificity associated with maltreatment but not autism. Detecting autism-related effects may require developing a new and sensitive method without needing such ambitious, labour-intensive, and potentially unfeasible sample size with these populations which can often be challenging to recruit.

Avoidant coping– assessed with a CRIES subscale– refers to strategies to avoid trauma-related information (Perrin et al., 2005). While more avoidant coping was associated with *less* specificity in the autism/TD group, consistent with a cognitive style for dampening painful details (Hitchcock et al., 2017), it was associated instead with *more* specificity (i.e., a better outcome) in the maltreatment group. This puzzling finding has been found in individuals with trauma histories (Kuyken et al., 2006) and high self-harm risks (Startup et al., 2001). A possible explanation is that avoidant coping is maladaptive for individuals with non-maltreatment adversity in the general population (as with the autism/TD groups) but adaptive (at least in the short-term) for those with complex/extreme adversities (e.g., requiring social care input as in most of our maltreatment sample). Longitudinal studies can explore if maltreatment-exposed YP transition to reduced specificity over time, while distinguishing adaptive versus non-adaptive coping. The potential adaptive nature of avoidance and reduced FT/memory specificity during childhood in a maltreatment sample is consistent with the notion of “latent vulnerability”, reflecting calibration of the cognitive system to a highly threatening environment (McCrory & Viding, 2015).

Executive functioning did not appear to underpin FT specificity in autism in our exploratory analyses. We used a questionnaire (DEX-C), rather than established cognitive tests that have previously shown associations with autobiographical memory specificity (e.g., verbal fluency; Dalgleish et al., 2007). This questionnaire, which focussed on behavioural impact, likely conflates multiple aspects of executive functioning, including planning, inhibitory control, and working memory (Miyake et al., 2000). Dissecting these different aspects will be important in future research. For example, as most measures– questionnaires or cognitive tests– are not “process pure”, one could use multiple tests/measures and model the common variance (e.g., Hughes et al., 2009).

Autistic/maltreatment-exposed YP and their families have indicated that improving mental health is their research priority (Richardson & Lelliott, 2003; Roche et al., 2021). Relevant to this call, promising associations were found between the specificity of future events (following voluntary generation) and depression, but not anxiety, as found with TD adults (Du et al., 2022; Gamble et al., 2019), and here extended to autistic individuals for the first time. Impaired generation of specific, personally-relevant future episodes could hinder adaptive emotional regulation, for example, to enable one to vividly envision an optimistic future outcome or to accurately assess negative predictions while limiting catastrophising. Thus, FT specificity may be relevant for (autistic) adolescents as a risk marker for depression prevention and/or treatment, especially during a developmental period of heightened cognitive malleability (Lau & Waters, 2017). Emerging low-intensity therapeutics, which have shown promise in “re-training” the specificity of autobiographical thinking with downstream effects on emotional symptoms in TD individuals (Hitchcock et al., 2017; Jing et al., 2016), could be explored in autistic/maltreatment-exposed populations. Note in this same youth sample, unlike depression, anxiety appeared to be more closely linked to features of *involuntary* generation of future events instead (see Lau-Zhu et al., 2024). More broadly, innovations for youth mental health (and vulnerable groups) can draw from clinical insights on emotional future thinking, which have primarily been focussed on adults so far (e.g., Di Simplicio et al., 2016, 2019; Lau-Zhu et al., 2023), especially as autistic adolescents overall appear to be able to imagine, contrary to existing assumptions (APA, 2013).

We acknowledge limitations due the resource constraints. We lacked direct diagnostic assessments (e.g., autism and emotional disorders). However, we did collect well-established measures and multi-informant data as much as possible (including verifications from keyworkers when possible within the autism/maltreatment group) while minimising caregiver burden during the peaks of the COVID-19 pandemic in the UK, when the data collection took place. Information on attachment was also not gathered, which is a supposed source of overlap between maltreatment and autism (Davidson et al., 2015; Moran, 2010) and associated with autobiographical thinking (Lau-Zhu, Williams et al., 2023). A group with both autism and maltreatment was not included to test for “double whammy/jeopardy” (Dinkler et al., 2017; Gajwani & Minnis, 2023). Inclusion of matched groups on anxiety/depression diagnoses could also be fruitful in future research to better understand the role of co-occurring emotional disorders.

Our measure of GCA tapped into nonverbal reasoning, so aspects of verbal reasoning such as verbal IQ, language skills, or narrative abilities could underlie some of the observed effects on FT and thus should also be assessed and adjusted for in future. Note however that unlike other FT tasks (e.g., Lind et al., 2014), ours only required short sentences rather than complex descriptions. A measure of home environment could be considered to account for potential distractions influencing cognitive performance. Additional tests could also help ascertain whether the maltreatment-related effects reflect a general cognitive impairment or a specific impairment in FT/autobiographical processing.

While our sample was appropriately powered for our primary hypotheses (i.e., to detect large group differences based on prior literature), a larger sample could address potential heterogeneity within groups. This includes in cognitive abilities within autism (Happé & Frith, 2020) and in age, for example in relation to executive functioning (Best & Miller, 2010) and social pressures to setting future goals (Nurmi, 1991). Transitioning into young adulthood can be more challenging for autistic people relative to their TD peers (Aydin et al., 2022; Capp et al., 2022; Lau-Zhu et al., 2019), likely influencing perception of their future selves. As over 90% of autistic adolescents were of White ethnicity (but 61–68% in the maltreatment and TD groups), future work should further consider the impact of ethnic diversity. By laying a firm foundation for critical next steps in disentangling presentations following autism and maltreatment, we pave the way for translating cognitive science for clinical benefits in youth, and across typical and atypical development.

## Electronic Supplementary Material

Below is the link to the electronic supplementary material.

Supplementary Material 1

## Footnotes

1 The term “autistic” (rather than with autism) is the preferred term in the autism community in the United Kingdom (Kenny et al., 2016).

2 We will use the term “maltreatment” because, unlike “developmental trauma”, it has a more widely-agreed definition capturing early experiences of abuse/neglect (Cicchetti & Toth, 2005; Gee et al., 2020) without being over-inclusive (e.g., poverty, discrimination, or bullying).

## References

[CR1] Akbari, M., Jamshidi, S., Seydavi, M., & Hallford, D. J. (2023). Functions of episodic future thinking: A validation and comparative study across individuals with normal versus pathological worry. *Applied Cognitive Psychology*. 10.1002/ACP.4148.

[CR2] Altman, D. G. (1991). *Practical Statistics for Medical Research*. Chapman and Hall.

[CR3] American Psychiatric Association. (1994). *Diagnostic and statistical Manual of Mental Disorder* (4th ed.). American Psychiatric Association.

[CR4] American Psychiatric Association. (2013). *Diagnostic and statistical Manual of Mental Disorder* (5th ed.). American Psychiatric Association.

[CR5] Aydin, Ü., Capp, S. J., Tye, C., Colvert, E., Lau-Zhu, A., Rijsdijk, F., Palmer, J., & McLoughlin, G. (2022). Quality of life, functional impairment and continuous performance task event-related potentials (ERPs) in young adults with ADHD and autism: A twin study. *JCPP Advances*, *2*(3), e12090. 10.1002/JCV2.12090. 37431386 10.1002/jcv2.12090 PMC10242939

[CR6] Baldwin, J. R., Reuben, A., Newbury, J. B., & Danese, A. (2019). Agreement between prospective and retrospective measures of Childhood Maltreatment: A systematic review and Meta-analysis. *JAMA Psychiatry*, *76*(6), 584–593. 10.1001/JAMAPSYCHIATRY.2019.0097. 30892562 10.1001/jamapsychiatry.2019.0097 PMC6551848

[CR7] Barry, T. J., Hallford, D. J., & Takano, K. (2021). Autobiographical memory impairments as a transdiagnostic feature of mental illness: A meta-analytic review of investigations into autobiographical memory specificity and overgenerality among people with psychiatric diagnoses. *Psychological Bulletin*, *147*(10), 1054–1074. 10.1037/BUL0000345. 34968086 10.1037/bul0000345

[CR8] Benarous, X., Guilé, J. M., Consoli, A., & Cohen, D. (2015). A systematic review of the evidence for impaired cognitive theory of mind in maltreated children. *Frontiers in Psychiatry*, *6*(JUL), 1. 10.3389/FPSYT.2015.00108. 26283975 10.3389/fpsyt.2015.00108 PMC4516890

[CR9] Best, J. R., & Miller, P. H. (2010). A developmental perspective on executive function. *Child Development*, *81*(6), 1641–1660. 10.1111/J.1467-8624.2010.01499.X. 21077853 10.1111/j.1467-8624.2010.01499.x PMC3058827

[CR10] Bilker, W. B., Hansen, J. A., Brensinger, C. M., Richard, J., Gur, R. E., & Gur, R. C. (2012). Development of abbreviated nine-item forms of the raven’s Standard Progressive matrices Test. *Assessment*, *19*(3), 354–369. 10.1177/1073191112446655. 22605785 10.1177/1073191112446655 PMC4410094

[CR11] Bone, J. K., Lewis, G., Roiser, J. P., Blakemore, S. J., & Lewis, G. (2021). Recall bias during adolescence: Gender differences and associations with depressive symptoms. *Journal of Affective Disorders*, *282*, 299–307. 10.1016/J.JAD.2020.12.133. 33421856 10.1016/j.jad.2020.12.133 PMC7615279

[CR13] Brown, G. P., MacLeod, A. K., Tata, P., & Goddard, L. (2002). Worry and the simulation of future outcomes. *Anxiety Stress & Coping*, *15*(1), 1–17. 10.1080/10615800290007254.

[CR12] Brown, A. D., Root, J. C., Romano, T. A., Chang, L. J., Bryant, R. A., & Hirst, W. (2013). Overgeneralized autobiographical memory and future thinking in combat veterans with posttraumatic stress disorder. *Journal of Behavior Therapy and Experimental Psychiatry*, *44*(1), 129–134. 10.1016/j.jbtep.2011.11.004. 22200095 10.1016/j.jbtep.2011.11.004

[CR14] Brunsdon, V. E. A., & Happé, F. (2013). Exploring the ‘fractionation’ of autism at the cognitive level. *Autism*, *18*(1), 17–30. 10.1177/1362361313499456. 24126870 10.1177/1362361313499456

[CR15] Capp, S. J., Agnew-Blais, J., Lau-Zhu, A., Colvert, E., Tye, C., Aydin, Ü., Lautarescu, A., Ellis, C., Saunders, T., O’Brien, L., Ronald, A., Happé, F., & McLoughlin, G. (2022). Is quality of life related to high autistic traits, high ADHD traits and their Interaction? Evidence from a Young-Adult Community-based twin sample. *Journal of Autism and Developmental Disorders*. 10.1007/S10803-022-05640-W. 35802291 10.1007/s10803-022-05640-w PMC10465683

[CR16] Chorpita, B. F., Moffitt, C. E., & Gray, J. (2005). Psychometric properties of the revised child anxiety and Depression Scale in a clinical sample. *Behaviour Research and Therapy*, *43*(3), 309–322. 10.1016/j.brat.2004.02.004. 15680928 10.1016/j.brat.2004.02.004

[CR17] Ciaramelli, E., Spoglianti, S., Bertossi, E., Generali, N., Telarucci, F., Tancredi, R., Muratori, F., & Igliozzi, R. (2018). Construction of past and future events in children and adolescents with ASD: Role of self-relatedness and relevance to decision-making. *Journal of Autism and Developmental Disorders*, *48*(9), 2995–3009. 10.1007/s10803-018-3577-y. 29644583 10.1007/s10803-018-3577-y

[CR18] Cicchetti, D., & Toth, S. L. (2005). Childhood maltreatment. *Annual Review of Clinical Psychology*, *1*(1), 409–438. 10.1146/annurev.clinpsy.1.102803.144029. 17716094 10.1146/annurev.clinpsy.1.102803.144029

[CR19] Coughlan, B., Woolgar, M., van Ijzendoorn, M. H., & Duschinsky, R. (2021). Socioemotional profiles of autism spectrum disorders, attention deficit hyperactivity disorder, and disinhibited and reactive attachment disorders: A symptom comparison and network approach. *Development and Psychopathology*, 1–10. 10.1017/S0954579421000882. 10.1017/S0954579421000882 34766900

[CR20] Crane, L., Lind, S. E., & Bowler, D. M. (2013). Remembering the past and imagining the future in autism spectrum disorder. *Memory (Hove, England)*, *21*(2), 157–166. 10.1080/09658211.2012.712976. 22901078 10.1080/09658211.2012.712976

[CR21] Cui, Z., Oshri, A., Liu, S., Smith, E. P., & Kogan, S. M. (2020). Child maltreatment and resilience: The promotive and protective role of future orientation. *Journal of Youth and Adolescence*, *49*(10), 2075–2089. 10.1007/S10964-020-01227-9/FIGURES/2. 32236791 10.1007/s10964-020-01227-9

[CR24] D’Argembeau, A., & Mathy, A. (2011). Tracking the construction of episodic future thoughts. *Journal of Experimental Psychology: General*, *140*(2), 258–271. 10.1037/A0022581. 21401291 10.1037/a0022581

[CR25] D’Argembeau, A., Renaud, O., & Van Der Linden, M. (2011). Frequency, characteristics and functions of future-oriented thoughts in daily life. *Applied Cognitive Psychology*, *25*(1), 96–103. 10.1002/ACP.1647.

[CR22] Dalgleish, T., Golden, A. M. J., Barrett, L. F., Yeung, A., Murphy, C., Tchanturia, V., Williams, K., Perkins, J. M. G., Barnard, N., Elward, P. J., R., & Watkins, E. (2007). Reduced specificity of autobiographical memory and depression: The role of executive control. *Journal of Experimental Psychology: General*, *136*(1), 23–42. 10.1037/0096-3445.136.1.23. 17324083 10.1037/0096-3445.136.1.23 PMC2225543

[CR23] Danese, A., Moffitt, T. E., Arseneault, L., Bleiberg, B. A., Dinardo, P. B., Gandelman, S. B., Houts, R., Ambler, A., Fisher, H. L., Poulton, R., & Caspi, A. (2017). The origins of cognitive deficits in victimized children: Implications for neuroscientists and clinicians. *American Journal of Psychiatry*, *174*(4), 349–361. 10.1176/APPI.AJP.2016.16030333/ASSET/IMAGES/LARGE/APPI.AJP.2016.16030333F3.JPEG. 27794691 10.1176/appi.ajp.2016.16030333 PMC5378606

[CR27] Davidson, C., O’Hare, A., Mactaggart, F., Green, J., Young, D., Gillberg, C., & Minnis, H. (2015). Social relationship difficulties in autism and reactive attachment disorder: Improving diagnostic validity through structured assessment. *Research in Developmental Disabilities*, *40*, 63–72. 10.1016/j.ridd.2015.01.007. 25754456 10.1016/j.ridd.2015.01.007

[CR26] Davidson, C., Moran, H., & Minnis, H. (2022). Autism and attachment disorders– how do we tell the difference? *BJPsych Advances*, 1–10. 10.1192/BJA.2022.2.

[CR29] Di Simplicio, M., Renner, F., Blackwell, S. E., Mitchell, H., Stratford, H. J., Watson, P., Myers, N., Nobre, A. C., Lau-Zhu, A., & Holmes, E. A. (2016). An investigation of mental imagery in bipolar disorder: Exploring the mind’s eye. *Bipolar Disorders*, *18*(8), 669–683. 10.1111/bdi.12453. 27995690 10.1111/bdi.12453 PMC5299482

[CR28] Di Simplicio, M., Lau-Zhu, A., Meluken, I., Taylor, P., Kessing, L. V., Vinberg, M., Holmes, E. A., & Miskowiak, K. W. (2019). Emotional mental imagery abnormalities in monozygotic twins with, at high-risk of, and without affective disorders: Present in affected twins in remission but absent in high-risk twins. *Frontiers in Psychiatry*, *10*(801), eCollection 2019. 10.3389/fpsyt.2019.00801. 10.3389/fpsyt.2019.00801 PMC6856790 31780967

[CR30] Dinkler, L., Lundström, S., Gajwani, R., Lichtenstein, P., Gillberg, C., & Minnis, H. (2017). Maltreatment-associated neurodevelopmental disorders: A co-twin control analysis. *Journal of Child Psychology and Psychiatry*, *58*(6), 691–701. 10.1111/JCPP.12682. 28094432 10.1111/jcpp.12682

[CR31] Du, J. Y., Hallford, D. J., & Busby Grant, J. (2022). Characteristics of episodic future thinking in anxiety: A systematic review and meta-analysis. *Clinical Psychology Review*, *95*, 102162. 10.1016/J.CPR.2022.102162. 35660923 10.1016/j.cpr.2022.102162

[CR32] Emslie, H., Wilson, F. C., Burden, V., Nimmo-Smith, I., & Wilson, B. A. (2003). *Behavioural Assessment of the Dysexecutive syndrome for children (BADS-C)*. Thames Valley Test Company.

[CR33] Feller, C., Dubois, C., Eliez, S., & Schneider, M. (2021). Episodic future thinking in autism spectrum disorder and 22q11.2 deletion Syndrome: Association with anticipatory pleasure and social functioning. *Journal of Autism and Developmental Disorders*, *51*(12), 4587–4604. 10.1007/S10803-021-04903-2/TABLES/7. 33586083 10.1007/s10803-021-04903-2 PMC8592949

[CR34] Ford, T., Vostanis, P., Meltzer, H., & Goodman, R. (2007). Psychiatric disorder among British children looked after by local authorities: Comparison with children living in private households. *British Journal of Psychiatry*, *190*(4), 319–325. 10.1192/BJP.BP.106.025023. 10.1192/bjp.bp.106.025023 17401038

[CR35] Frith, U. (2001). What framework should we use for understanding developmental disorders? *Developmental Neuropsychology*, *20*(2), 555–563. 10.1207/S15326942DN2002_6. 11892952 10.1207/S15326942DN2002_6

[CR36] Fritz, C. O., Morris, P. E., & Richler, J. J. (2012). Effect size estimates: Current use, calculations, and interpretation. *Journal of Experimental Psychology: General*, *141*(1), 2–18. 10.1037/A0024338. 21823805 10.1037/a0024338

[CR38] Gajwani, R., & Minnis, H. (2023). Double jeopardy: Implications of neurodevelopmental conditions and adverse childhood experiences for child health. *European Child & Adolescent Psychiatry*, *32*(1), 1. 10.1007/S00787-022-02081-9. 36156745 10.1007/s00787-022-02081-9 PMC9908716

[CR37] Gajwani, R., Dinkler, L., Lundström, S., Lichtenstein, P., Gillberg, C., & Minnis, H. (2021). Mania symptoms in a Swedish longitudinal population study: The roles of childhood trauma and neurodevelopmental disorders. *Journal of Affective Disorders*, *280*, 450–456. 10.1016/J.JAD.2020.10.076. 33242716 10.1016/j.jad.2020.10.076

[CR39] Gamble, B., Moreau, D., Tippett, L. J., & Addis, D. R. (2019). Specificity of future thinking in depression: A meta-analysis. *Perspectives on Psychological Science*, *14*(5), 816–834. 10.1177/1745691619851784. 31374179 10.1177/1745691619851784

[CR40] Gee, D., Colich, N., Sheridan, M., Pine, D., & McLaughlin, K. (2020). Youth exposed to Maltreatment Show Age-related alterations in hippocampal-fronto-amygdala function during extinction recall. *Biological Psychiatry*, *87*(9), S104. 10.1016/j.biopsych.2020.02.284.

[CR41] Gilbert, R., Widom, C. S., Browne, K., Fergusson, D., Webb, E., & Janson, S. (2009). Burden and consequences of child maltreatment in high-income countries. *The Lancet*, *373*(9657), 68–81. 10.1016/S0140-6736(08)61706-7. 10.1016/S0140-6736(08)61706-7 19056114

[CR42] Gillberg, C., & Billstedt, E. (2000). Autism and Asperger syndrome: Coexistence with other clinical disorders. *Acta Psychiatrica Scandinavica*, *102*(5), 321–330. 10.1034/J.1600-0447.2000.102005321.X. 11098802 10.1034/j.1600-0447.2000.102005321.x

[CR43] Goddard, L., Dritschel, B., Robinson, S., & Howlin, P. (2014). Development of autobiographical memory in children with autism spectrum disorders: Deficits, gains, and predictors of performance. *Development and Psychopathology*, *26*(1), 215–228. 10.1017/S0954579413000904. 24284059 10.1017/S0954579413000904

[CR44] Goodman, G. S., Quas, J. A., & Ogle, C. M. (2010). Child maltreatment and memory. *Annual Review of Psychology*, *61*, 325–351. 10.1146/ANNUREV.PSYCH.093008.100403. 19575622 10.1146/annurev.psych.093008.100403

[CR45] Hallford, D. J., Austin, D. W., Takano, K., & Raes, F. (2018). Psychopathology and episodic future thinking: A systematic review and meta-analysis of specificity and episodic detail. *Behaviour Research and Therapy*, *102*, 42–51. 10.1016/J.BRAT.2018.01.003. 29328948 10.1016/j.brat.2018.01.003

[CR46] Happé, F., & Frith, U. (2006). The weak coherence account: Detail-focused cognitive style in autism spectrum disorders. *Journal of Autism and Developmental Disorders*, *36*(1), 5–25. 10.1007/s10803-005-0039-0. 16450045 10.1007/s10803-005-0039-0

[CR47] Happé, F., & Frith, U. (2020). Annual Research Review: Looking back to look forward– changes in the concept of autism and implications for future research. *Journal of Child Psychology and Psychiatry*, *61*(3), 218–232. 10.1111/JCPP.13176. 31994188 10.1111/jcpp.13176

[CR48] Harvey, A. G., Watkins, E., Mansell, W., & Shafran, R. (2004). *Cognitive behavioural processes across psychological disorders: A Transdiagnostic Approach to Research and Treatment* (1st ed.). Oxford University Press.

[CR49] Heron, J., Crane, C., Gunnell, D., Lewis, G., Evans, J., & Williams, J. M. G. (2012). 40,000 memories in young teenagers: Psychometric properties of the autobiographical memory test in a UK cohort study. *Memory (Hove, England)*, *20*(3), 320. 10.1080/09658211.2012.656846. 10.1080/09658211.2012.656846 PMC3379787 22348421

[CR50] Hitchcock, C., Nixon, R. D. V., & Weber, N. (2014). A review of overgeneral memory in child psychopathology. *British Journal of Clinical Psychology*, *53*(2), 170–193. 10.1111/bjc.12034. 24921070 10.1111/bjc.12034

[CR51] Hitchcock, C., Werner-Seidler, A., Blackwell, S. E., & Dalgleish, T. (2017). Autobiographical episodic memory-based training for the treatment of mood, anxiety and stress-related disorders: A systematic review and meta-analysis. *Clinical Psychology Review*, *52*, 92–107. 10.1016/J.CPR.2016.12.003. 28086133 10.1016/j.cpr.2016.12.003

[CR52] Hoover, D. W. (2015). The effects of psychological trauma on children with autism spectrum disorders: A research review. *Review Journal of Autism and Developmental Disorders*, *2*(3), 287–299. 10.1007/s40489-015-0052-y.

[CR53] Hughes, C., Ensor, R., Wilson, A., & Graham, A. (2009). Tracking executive function across the transition to school: A latent variable approach. *Developmental Neuropsychology*, *35*(1), 20–36. 10.1080/87565640903325691. 10.1080/87565640903325691 20390590

[CR54] IBM (2020). *IBM SPSS Statistics 27*.

[CR56] Ji, J. L., Kavanagh, D. J., Holmes, E. A., MacLeod, C., & Simplicio, D., M (2019). Mental imagery in psychiatry: Conceptual and clinical implications. *CNS Spectrums*, *24*(1), 114–126. 10.1017/S1092852918001487. 30688194 10.1017/S1092852918001487

[CR55] Ji, J. L., Geiles, D., & Saulsman, L. M. (2021). Mental imagery-based episodic simulation amplifies motivation and behavioural engagement in planned reward activities. *Behaviour Research and Therapy*, *145*. 10.1016/J.BRAT.2021.103947. 10.1016/j.brat.2021.103947 34433114

[CR57] Jing, H. G., Madore, K. P., & Schacter, D. L. (2016). Worrying about the future: An episodic specificity induction impacts problem solving, reappraisal, and well-being. *Journal of Experimental Psychology: General*, *145*(4), 402. 10.1037/XGE0000142. 26820166 10.1037/xge0000142 PMC4792686

[CR58] Jing, H. G., Madore, K. P., & Schacter, D. L. (2019). Not to worry: Episodic retrieval impacts emotion regulation in older adults. *Emotion*. 10.1037/EMO0000581. 30816741 10.1037/emo0000581 PMC6715543

[CR59] Johnson, S. R. L., Blum, R. W., & Cheng, T. L. (2014). Future orientation: A construct with implications for adolescent health and wellbeing. *International Journal of Adolescent Medicine and Health*, *26*(4), 468. 10.1515/IJAMH-2013-0333. 10.1515/ijamh-2013-0333 PMC4827712 24523304

[CR60] Kaufman, J., Jones, B., Stieglitz, E., Vitulano, L., & Mannarino, A. P. (1994). The use of multiple informants to assess children’s maltreatment experiences. *Journal of Family Violence*, *9*(3), 227–248. 10.1007/BF01531949.

[CR61] Kenny, L., Hattersley, C., Molins, B., Buckley, C., Povey, C., & Pellicano, E. (2016). Which terms should be used to describe autism? Perspectives from the UK autism community. *Autism*, *20*(4), 442–462. 10.1177/1362361315588200. 26134030 10.1177/1362361315588200

[CR62] Kleim, B., Graham, B., Fihosy, S., Stott, R., & Ehlers, A. (2014). Reduced specificity in episodic future thinking in posttraumatic stress disorder. *Clinical Psychological Science*, *2*(2), 165–173. 10.1177/2167702613495199. 24926418 10.1177/2167702613495199 PMC4051242

[CR63] Kuyken, W., Howell, R., & Dalgleish, T. (2006). Overgeneral autobiographical memory in depressed adolescents with, versus without, a reported history of trauma. *Journal of Abnormal Psychology*, *115*(3), 387–396. 10.1037/0021-843X.115.3.387. 16866580 10.1037/0021-843X.115.3.387

[CR64] Lacey, R. E., & Minnis, H. (2020). Practitioner review: Twenty years of research with adverse childhood experience scores– advantages, disadvantages and applications to practice. *Journal of Child Psychology and Psychiatry*, *61*(2), 116–130. 10.1111/JCPP.13135. 31609471 10.1111/jcpp.13135

[CR65] Lai, M. C., Kassee, C., Besney, R., Bonato, S., Hull, L., Mandy, W., Szatmari, P., & Ameis, S. H. (2019). Prevalence of co-occurring mental health diagnoses in the autism population: A systematic review and meta-analysis. *The Lancet Psychiatry*, *6*(10), 819–829. 10.1016/S2215-0366(19)30289-5. 31447415 10.1016/S2215-0366(19)30289-5

[CR66] Lau, J. Y. F., & Waters, A. M. (2017). Annual Research Review: An expanded account of information-processing mechanisms in risk for child and adolescent anxiety and depression. *Journal of Child Psychology and Psychiatry and Allied Disciplines*, *58*(4), 387–407. 10.1111/JCPP.12653. 27966780 10.1111/jcpp.12653

[CR70] Lau-Zhu, A., & Vella, L. (2023). A Compassion-focused therapy group for young people who live in foster, adoptive or kinship care: Initial development, reflections, and ways forward. *Adoption & Fostering*. 10.1177/03085759231207397.

[CR67] Lau-Zhu, A., Fritz, A., & McLoughlin, G. (2019). Overlaps and distinctions between attention-deficit/hyperactivity disorder and autism spectrum disorder in young adulthood: A systematic review and guiding framework for EEG-imaging research. *Neuroscience and Biobehavioral Reviews*, *96*, 93–115. 30367918 10.1016/j.neubiorev.2018.10.009 PMC6331660

[CR71] Lau-Zhu, A., Williams, F., & Steel, C. (2023). Attachment patterns and autobiographical episodic memory functioning: A systemic review of adult studies to advance clinical psychological science. *Clinical Psychology Review*, *101*, 102254. 10.1016/J.CPR.2023.102254. 36804184 10.1016/j.cpr.2023.102254

[CR69] Lau-Zhu, A., Tuxen, N., Roerne, M. L., & Di Simplicio, M. (2023a). Imagery-based cognitive therapy for comorbid anxiety in bipolar disorder: Two case studies in Denmark. *Psychiatry Research Case Reports*, *2*(1), 100124. 10.1016/J.PSYCR.2023.100124.

[CR68] Lau-Zhu, A., Stacey, J., Gibson, D., Chan, C., & Cooper, M. (2024). Flashforward mental imagery in adolescents: Exploring developmental differences and associations with mental health. *Behavioural and Cognitive Psychotherapy, Accepted*. 10.1017/S1352465824000298 PMC7618818 39308216

[CR72] Liberatos, P., Link, B. G., & Kelsey, J. L. (1988). The measurement of social class in epidemiology. *Epidemiologic Reviews*, *10*(1), 87–121. 10.1093/OXFORDJOURNALS.EPIREV.A036030. 3066632 10.1093/oxfordjournals.epirev.a036030

[CR73] Lind, S. E., & Bowler, D. M. (2010). Episodic memory and episodic future thinking in adults with autism. *Journal of Abnormal Psychology*, *119*(4), 896–905. 10.1037/a0020631. 20853917 10.1037/a0020631

[CR74] Lind, S. E., Williams, D. M., Bowler, D. M., & Peel, A. (2014). Episodic memory and episodic future thinking impairments in high-functioning autism spectrum disorder: An underlying difficulty with scene construction or self-projection? *Neuropsychology*, *28*(1), 55–67. 10.1037/neu0000005. 24015827 10.1037/neu0000005 PMC3906795

[CR75] Lippard, E. T. C., & Nemeroff, C. B. (2020). The devastating clinical consequences of child abuse and neglect: Increased disease vulnerability and poor treatment response in mood disorders. *American Journal of Psychiatry*, *177*(1), 20–36. 10.1176/appi.ajp.2019.19010020. 31537091 10.1176/appi.ajp.2019.19010020 PMC6939135

[CR76] Maccallum, F., & Bryant, R. A. (2011). Imagining the future in complicated grief. *Depression and Anxiety*, *28*(8), 658–665. 10.1002/da.20866. 21796741 10.1002/da.20866

[CR77] May-Chahal, C., & Cawson, P. (2005). Measuring child maltreatment in the United Kingdom: A study of the prevalence of child abuse and neglect. *Child Abuse and Neglect*, *29*(9), 969–984. 10.1016/j.chiabu.2004.05.009. 16165212 10.1016/j.chiabu.2004.05.009

[CR79] McCrory, E. J., & Viding, E. (2015). The theory of latent vulnerability: Reconceptualizing the link between childhood maltreatment and psychiatric disorder. *Development and Psychopathology*, *27*(2), 493–505. 10.1017/S0954579415000115. 25997767 10.1017/S0954579415000115

[CR78] McCrory, E. J., Puetz, V. B., Maguire, E. A., Mechelli, A., Palmer, A., Gerin, M. I., Kelly, P. A., Koutoufa, I., & Viding, E. (2017). Autobiographical memory: A candidate latent vulnerability mechanism for psychiatric disorder following childhood maltreatment. *British Journal of Psychiatry*, *211*(4), 216–222. 10.1192/bjp.bp.117.201798. 10.1192/bjp.bp.117.201798 PMC5623877 28882830

[CR81] McDonnell, C. G., Valentino, K., & Diehl, J. J. (2017). A developmental psychopathology perspective on autobiographical memory in autism spectrum disorder. *Developmental Review*, *44*, 59–81. 10.1016/j.dr.2017.01.001.

[CR80] McDonnell, C. G., Boan, A. D., Bradley, C. C., Seay, K. D., Charles, J. M., & Carpenter, L. A. (2019). Child maltreatment in autism spectrum disorder and intellectual disability: Results from a population-based sample. *Journal of Child Psychology and Psychiatry*, *60*(5), 576–584. 10.1111/JCPP.12993. 30368827 10.1111/jcpp.12993 PMC6458088

[CR82] McKenzie, R., & Dallos, R. (2017). Autism and attachment difficulties: Overlap of symptoms, implications and innovative solutions. *Clinical Child Psychology and Psychiatry*, *22*(4), 632–648. 10.1177/1359104517707323. 28530116 10.1177/1359104517707323

[CR83] Meltzer, H., Corbin, T., Gatward, R., Goodman, R., & Ford, T. (2003). *The Mental Health of Young People Looked-after by Local Authorities in England*. HMSO.

[CR84] Minnis, H., Messow, C. M., McConnachie, A., Bradshaw, P., Briggs, A., Wilson, P., Gillberg, C., & Health, M. (2020). Autism and attachment disorder symptoms in the general population: Prevalence, overlap, and burden. *Developmental Child Welfare*, *2*(1), 37–51. 10.1177/2516103220902778. 10.1177/2516103220902778 PMC13021084 41907538

[CR85] Miyake, A., Friedman, N. P., Emerson, M. J., Witzki, A. H., Howerter, A., & Wager, T. D. (2000). The unity and diversity of executive functions and their contributions to complex frontal lobe tasks: A latent variable analysis. *Cognitive Psychology*, *41*(1), 49–100. 10.1006/COGP.1999.0734. 10945922 10.1006/cogp.1999.0734

[CR86] Moore, S. A., & Zoellner, L. A. (2007). Overgeneral autobiographical memory and traumatic events: An evaluative review. *Psychological Bulletin*, *133*(3), 419–437. 10.1037/0033-2909.133.3.419. 17469985 10.1037/0033-2909.133.3.419 PMC2665927

[CR87] Moran, H. J. (2010). Clinical observations of the differences between children in the autism spectrum and those with attachment problems: The Coventry Grid. *Good Autism Practice*, *11*(2), 46–59.

[CR88] Morin, J. F. G., Afzali, M. H., Bourque, J., Stewart, S. H., Séguin, J. R., O’Leary-Barrett, M., & Conrod, P. J. (2019). A population-based analysis of the relationship between substance use and adolescent cognitive development. *American Journal of Psychiatry*, *176*(2), 98–106. 10.1176/APPI.AJP.2018.18020202/ASSET/IMAGES/LARGE/APPI.AJP.2018.18020202F2.JPEG. 30278790 10.1176/appi.ajp.2018.18020202

[CR89] Nurmi, J. E. (1991). How do adolescents see their future? A review of the development of future orientation and planning. *Developmental Review*, *11*(1), 1–59. 10.1016/0273-2297(91)90002-6.

[CR90] O’Hearn, K., Asato, M., Ordaz, S., & Luna, B. (2008). Neurodevelopment and executive function in autism. *Development and Psychopathology*, *20*(04), 1103. 10.1017/S0954579408000527. 18838033 10.1017/S0954579408000527

[CR91] Perrin, S., Meiser-Stedman, R., & Smith, P. (2005). The children’s revised impact of event scale (CRIES): Validity as a screening instrument for PTSD. *Behavioural and Cognitive Psychotherapy*, *33*(4), 487–498. 10.1017/S1352465805002419.

[CR92] Pind, J., Gunnardsdóttir, E. K., & Jóhannesson, H. S. (2003). Raven’s Standard Progressive Matrices: New school age norms and a study of the test’s validity. *Personality and Individual Differences*, *34*(3), 375–386. 10.1016/S0191-8869(02)00058-2.

[CR93] Qualtrics. (2020). *Qualtrics*. Qualtrics.

[CR94] Radez, J., Waite, P., Chorpita, B., Creswell, C., Orchard, F., Percy, R., Spence, S. H., & Reardon, T. (2021). Using the 11-item version of the RCADS to identify anxiety and depressive disorders in adolescents. *Research on Child and Adolescent Psychopathology*, *49*(9), 57. 10.1007/S10802-021-00817-W. 10.1007/s10802-021-00817-w PMC8321965 33792821

[CR122] Raven, J. (2000). The Raven’s progressive matrices: Change and stability over culture and time. *Cognitive Psychology, 41*, 1–48. 10.1006/cogp.1999.0735 10.1006/cogp.1999.0735 10945921

[CR95] Richardson, J., & Lelliott, P. (2003). Mental health of looked after children. *Advances in Psychiatric Treatment*, *9*(4), 249–256. 10.1192/APT.9.4.249.

[CR96] Robinaugh, D. J., & McNally, R. J. (2013). Remembering the past and envisioning the future in bereaved adults with and without complicated grief. *Clinical Psychological Science*, *1*(3), 290–300. 10.1177/2167702613476027.

[CR97] Roche, L., Adams, D., & Clark, M. (2021). Research priorities of the autism community: A systematic review of key stakeholder perspectives. *Autism*, *25*(2), 336–348. 10.1177/1362361320967790. 33143455 10.1177/1362361320967790

[CR98] Roiser, J. P., & Sahakian, B. J. (2013). Hot and cold cognition in depression. *CNS Spectrums*, *18*(3), 139–149. 10.1017/S1092852913000072. 23481353 10.1017/S1092852913000072

[CR99] Rutter, M., Bailey, A., & Lord, C. (2003). *The Social Communication Questionnaire*. Western Psychological Services.

[CR100] Rutter, M., Kreppner, J., Croft, C., Murin, M., Colvert, E., Beckett, C., Castle, J., & Sonuga-Barke, E. (2007). Early adolescent outcomes of institutionally deprived and non-deprived adoptees. III. Quasi-autism. *Journal of Child Psychology and Psychiatry and Allied Disciplines*, *48*(12), 1200–1207. 10.1111/j.1469-7610.2007.01792.x. 18093025 10.1111/j.1469-7610.2007.01792.x

[CR101] Sachser, C., Berliner, L., Holt, T., Jensen, T. K., Jungbluth, N., Risch, E., Rosner, R., & Goldbeck, L. (2017). International development and psychometric properties of the child and adolescent trauma screen (CATS). *Journal of Affective Disorders*, *210*, 189–195. 10.1016/j.jad.2016.12.040. 28049104 10.1016/j.jad.2016.12.040

[CR102] Schacter, D. L., & Addis, D. R. (2007). The cognitive neuroscience of constructive memory: Remembering the past and imagining the future. *Philosophical Transactions of the Royal Society of London Series B Biological Sciences*, *362*(1481), 773–786. 10.1098/rstb.2007.2087. 17395575 10.1098/rstb.2007.2087 PMC2429996

[CR103] Schacter, D. L., Benoit, R. G., & Szpunar, K. K. (2017). Episodic future thinking: Mechanisms and functions. *Current Opinion in Behavioral Sciences*, *17*, 41–50. 10.1016/j.cobeha.2017.06.002. 29130061 10.1016/j.cobeha.2017.06.002 PMC5675579

[CR104] Spain, D., Sin, J., Linder, K. B., McMahon, J., & Happé, F. (2018). Social anxiety in autism spectrum disorder: A systematic review. *Research in Autism Spectrum Disorders*, *52*, 51–68. 10.1016/j.rasd.2018.04.007.

[CR105] Startup, M., Heard, H., Swales, M., Jones, B., Williams, J. M. G., & Jones, R. S. P. (2001). Autobiographical memory and parasuicide in borderline personality disorder. *British Journal of Clinical Psychology*, *40*(2), 113–120. 10.1348/014466501163535. 11446233 10.1348/014466501163535

[CR106] Steinberg, L., Graham, S., O’Brien, L., Woolard, J., Cauffman, E., & Banich, M. (2009). Age differences in future orientation and delay discounting. *Child Development*, *80*(1), 28–44. 10.1111/J.1467-8624.2008.01244.X. 19236391 10.1111/j.1467-8624.2008.01244.x

[CR107] Sumner, J. A., Griffith, J. W., & Mineka, S. (2010). Overgeneral autobiographical memory as a predictor of the course of depression: A meta-analysis. *Behaviour Research and Therapy*, *48*(7), 614–625. 10.1016/J.BRAT.2010.03.013. 20399418 10.1016/j.brat.2010.03.013 PMC2878838

[CR108] Szpunar, K. K., Spreng, R. N., & Schacter, D. L. (2014). A taxonomy of prospection: Introducing an organizational framework for future-oriented cognition. *Proceedings of the National Academy of Sciences of the United States of America*, *111*(52), 18414–18421. 10.1073/pnas.1417144111. 25416592 10.1073/pnas.1417144111 PMC4284580

[CR109] Tager-Flusberg, H. (2007). Evaluating the theory-of-mind hypothesis of autism. *Current Directions in Psychological Science*, *16*(6), 311–315. 10.1111/J.1467-8721.2007.00527.X.

[CR110] Takano, K., Mori, M., Nishiguchi, Y., Moriya, J., & Raes, F. (2017). Psychometric properties of the written version of the autobiographical memory test in a Japanese community sample. *Psychiatry Research*, *248*, 56–63. 10.1016/J.PSYCHRES.2016.12.019. 28013087 10.1016/j.psychres.2016.12.019

[CR111] Terrett, G., Rendell, P. G., Raponi-Saunders, S., Henry, J. D., Bailey, P. E., & Altgassen, M. (2013). Episodic future thinking in children with autism spectrum disorder. *Journal of Autism and Developmental Disorders*, *43*(11), 2558–2568. 10.1007/s10803-013-1806-y. 23504377 10.1007/s10803-013-1806-y

[CR112] Tick, B., Bolton, P., Happé, F., Rutter, M., & Rijsdijk, F. (2016). Heritability of autism spectrum disorders: A meta-analysis of twin studies. *Journal of Child Psychology and Psychiatry and Allied Disciplines*, *57*(5), 585–595. 10.1111/jcpp.12499. 26709141 10.1111/jcpp.12499 PMC4996332

[CR113] Uljarevic, M., & Hamilton, A. (2013). Recognition of emotions in autism: A formal meta-analysis. *Journal of Autism and Developmental Disorders*, *43*(7), 1517–1526. 10.1007/S10803-012-1695-5/FIGURES/3. 23114566 10.1007/s10803-012-1695-5

[CR114] Valentino, K., Toth, S. L., & Cicchetti, D. (2009). Autobiographical memory functioning among abused, neglected, and nonmaltreated children: The overgeneral memory effect. *Journal of Child Psychology and Psychiatry and Allied Disciplines*, *50*(8), 1029–1038. 10.1111/j.1469-7610.2009.02072.x. 19490313 10.1111/j.1469-7610.2009.02072.x PMC3513357

[CR115] Vrielynck, N., Deplus, S., & Philippot, P. (2007). Overgeneral autobiographical memory and depressive disorder in children. *Journal of Clinical Child and Adolescent Psychology*, *36*(1), 95–105. 10.1080/15374410709336572. 17206885 10.1080/15374410709336572

[CR116] Weston, L., Hodgekins, J., & Langdon, P. E. (2016). Effectiveness of cognitive behavioural therapy with people who have autistic spectrum disorders: A systematic review and meta-analysis. *Clinical Psychology Review*, *49*, 41–54. 10.1016/J.CPR.2016.08.001. 27592496 10.1016/j.cpr.2016.08.001

[CR117] Wiley, A. R., Rose, A. J., Burger, L. K., & Miller, P. J. (1998). Constructing autonomous selves through narrative practices: A comparative study of working-class and middle-class families. *Child Development*, *69*(3), 833–847. 10.1111/J.1467-8624.1998.TB06246.X.

[CR118] Williams, J. M. G., Nurs, N., Tyers, C., Rose, G., & MacLeod, A. K. (1996). The specificity of autobiographical memory and imageability of the future. *Memory & Cognition*, *24*(1), 116–125. 8822164 10.3758/bf03197278

[CR119] World Health Organization (2016). *International statistical classification of diseases and related health problems (10th ed.)*. https://icd.who.int/browse10/2016/en. 10.2471/BLT.25.294650 PMC13531092 42683092

[CR120] World Health Organization (2019). *International statistical classification of diseases and related health problems (11th ed.)*. https://icd.who.int/. 10.2471/BLT.25.294650 PMC13531092 42683092

[CR121] Yeung, M. K. (2022). A systematic review and meta-analysis of facial emotion recognition in autism spectrum disorder: The specificity of deficits and the role of task characteristics. *Neuroscience and Biobehavioral Reviews*, *133*. 10.1016/J.NEUBIOREV.2021.104518. 10.1016/j.neubiorev.2021.104518 34974069

