# Supplementary material for: Specificity of Episodic Future Thinking in Adolescents: Comparing Childhood Maltreatment, Autism Spectrum, and Typical Development
Source: Res Child Adolesc Psychopathol. 2024 Aug 21;52(11):1781–95. doi: 10.1007/s10802-024-01232-7 (PMC11564263; doi:10.1007/s10802-024-01232-7)
Supplement: Supplementary file 1 — Supplementary Material 1 [file 10802_2024_1232_MOESM1_ESM.docx]

**Supplemental Materials: Specificity of Episodic Future Thinking in Adolescents: Comparing Childhood Maltreatment, Autism Spectrum, and Typical Development**

**Additional Methods - Questionnaires**

***Revised Children’s Anxiety and Depression Scale – Youth Report 11 items*** (RCADS-11; Radez et al., 2021)

This 11-item self-report questionnaire is based on the original RCADS-47 (Chorpita, Moffitt, & Gray, 2005) and has been designed for screening clinically-significant anxiety and depression. The items are scored on a 4-point Likert scale (anchored by “never”, “sometimes”, “often”, and “always”). Total scores range from 0-33, with higher scores indicating more severity. It has good convergent/divergent validity (Radez et al., 2021). The suggested cut-off scores vary by sex (boys: anxiety ≥ 5, depression ≥ 8, total ≥ 9; girls: anxiety ≥ 9, depression ≥ 9, total ≥ 14), with sensitivity/specificity > .70 (Radez et al., 2021). Internal consistency (Cronbach’s alpha) in this study was good for both anxiety (overall = 0.85; autism = 0.81; maltreatment = 0.89; typical development or TD = 0.76) and depression subscales (overall = 0.86; autism = 0.87; maltreatment = 0.88; TD = 0.71).

***Children’s Revised Impact of Event Scale*** (CRIES; Perrin, Meiser-Stedman, & Smith, 2005)

This 8-item self-report questionnaire is a brief screening measure of post-traumatic stress disorder (PTSD) in young people aged 8-18. Each item is anchored by 4 response options (“not at all”, “rarely”, “sometimes”, “often”). Total scores range 0-40, with higher scores indicating more PTSD symptoms. It has good test re-test reliability (Deeba, Rapee, & Prvan, 2014; Verlinden et al., 2014), convergent validity (Deeba et al., 2014), and concurrent validity (Giannopoulou et al., 2006). The cut-off score is ≥ 17 for probable PTSD, with sensitivity of .94 and specificity of .59 in clinical samples (Perrin et al., 2005). Internal consistency (Cronbach’s alpha) in this study was good to excellent (overall = 0.92; autism = 0.89; maltreatment = 0.93; TD = 0.90).

***Revised Children’s Anxiety and Depression Scale – Parent Report 47 items*** (Chorpita et al., 2005)

This 47-item questionnaire is designed for assessing symptoms of anxiety and depression in young people aged 8-18 based on parent/caregiver reports. It has six subscales: separation anxiety disorder, social phobia, generalized anxiety disorder, panic disorder, obsessive compulsive disorder, and low mood (major depressive disorder). The items are scored on a 4-point Likert scale (anchored by “never”, “sometimes”, “often”, and “always”). It has good test-retest reliability (Chorpita, Yim, Moffitt, Umemoto, & Francis, 2000), convergent validity (Esbjørn, Sømhovd, Turnstedt, & Reinholdt-Dunne, 2012) and concurrent validity (Chorpita et al., 2005). Total scores are converted to *t*-scores based on normative data with a clinical cut-off of t ≥ 70 and with sensitivity/specificity > .70 (Chorpita et al., 2000). Internal consistency (Cronbach’s alpha) in this study was excellent for the overall anxiety subscale (overall = 0.96; autism = 0.95; maltreatment = 0.95; TD = 0.92) and acceptable to excellent for the depression subscale (overall = 0.90; autism = 0.88; maltreatment = 0.82; TD = 0.68).

***Child and Adolescent Trauma Screen – Caregiver*** (CATS; Sachser et al., 2017)

This questionnaire is designed for assessing symptoms of PTSD in young people aged 7-17 based on parental/caregiver reports. It has 15-items assessing potentially traumatic events and 20-items (response choices: “yes” or “no”) measuring PTSD symptoms (response choices: 4-point Likert scale anchored by “never”, “once in a while”, “half the time” and “almost always), based on DSM-5 criteria. Total scores range 0-60, with higher scores indicating more PTSD symptoms. It has good convergent/discriminant validity, construct validity, and test re-test reliability (Sachser et al., 2017). The cut-off score is ≥ 21 for probable PTSD, with sensitivity of .86 and specificity of .74 (Nilsson et al., 2020). Internal consistency (Cronbach’s alpha) in this study was good to excellent (overall = 0.94; autism = 0.87; maltreatment = 0.96; TD = 0.93).

***Social Communication Questionnaire – Current form*** (SCQ; Rutter, Bailey, & Lord, 2003)

This is a 40-item screening questionnaire for autism spectrum disorders (ASD) in childhood based on the Autism Diagnostic Interview-Revised (Lord, Rutter, & Le Couteur, 1994). It contains questions regarding the child’s social communication and language development over the most recent three-month period. This version was chosen rather the Lifetime form because we expected many caregivers (e.g., foster carers, adoptive families) of maltreated adolescents to not know in detail the adolescent’s developmental history prior to coming into their care. Total scores range 0-39. This measure has good discriminant validity (Berument, Rutter, Lord, Pickles, & Bailey, 1999; Chandler et al., 2007). The cut-off score of ≥ 15 suggests the need for further assessment of ASD, with sensitivity/specificity > .70 (Chesnut et al., 2017). Internal consistency (Cronbach’s alpha) in this study was acceptable to good (overall = 0.89; autism = 0.77; maltreatment = 0.76; TD = 0.73).

***Dysexecutive Questionnaire – Children*** (DEX-C; Emslie, Wilson, Burden, Nimmo-Smith, & Wilson, 2003)

This is a 20-item questionnaire probing for four domains of difficulties associated with executive functioning: emotional/personality, motivational, behavioural, and cognitive. It is to be completed by an adult who knows the child well. The items are scored on a 5-point Likert scale with scores ranging 0-4 (from “never” to “very often”). DEX-C scores correlate significantly with test scores on the Behavioural Assessment of the Dysexecutive Syndrome in Children (Emslie et al., 2003). Internal consistency (Cronbach’s alpha) in this study was acceptable to excellent (overall = 0.95; autism = 0.90; maltreatment = 0.93; TD = 0.73).

**Additional Results – Primary Analyses**

Restricting findings to *positive* cue words only, FT specificity scores were significantly lower in the maltreatment group compared to the TD group (*U* = 229.00, *Z* = 2.73, *p* = .006). There were no significant differences between the autism and TD groups (*U* = 321.00, *Z* = 1.28, *p* = .167), or the autism and maltreatment groups (*U* = 341.00, *Z* = 1.08, *p* = .282). Similarly, restricting findings to *negative* cue words only, FT specificity scores were significantly lower in the maltreatment group compared to the TD group (*U* = 231.00, *Z* = 2.72, *p* = .007). There were no significant differences between the autism and TD groups (*U* = 311.00, *Z* = 1.55, *p* = .121), or the autism and maltreatment groups (*U* = 341.00, *Z* = 1.09, *p* = .277).

**Additional Results – Sensitivity Analyses for Primary Group Comparisons**

***Removing Adolescents with Declared Emotional Disorders***

The remaining participants per group were autism (*n* = 21), maltreatment (*n* = 26), and TD (*n* = 28). FT specificity scores were significantly lower in the maltreatment group compared to the TD group (*U* = 194.50, *Z* = 2.96, *p* = .003). There were no significant differences between the autism and TD groups (*U* = 204.00, *Z* = 1.83, *p* = .067), or the autism and maltreatment groups (*U* = 241.50, *Z* = 0.68, *p* = .485).

***Removing Adolescents with Declared Neurodevelopmental Disorders***

The remaining participants per group were autism (*n* = 16), maltreatment (*n* = 26), and TD (*n* = 28). FT specificity scores were significantly lower in the maltreatment group compared to the TD group (*U* = 194.50, *Z* = 2.96, *p* = .003). There were no significant differences between the autism and TD groups (*U* = 178.00, *Z* = 1.13, *p* = .259), or the autism and maltreatment groups (*U* = 172.50, *Z* = 0.93, *p* = .351).

***Removing Adolescents Meeting Clinical Cut-off in RCADS-47 Overall T-scores (Caregiver Reports)***

The remaining participants per group were autism (*n* = 10), maltreatment (*n* = 11), and TD (*n* = 27). FT specificity scores were significantly lower (one-tailed) in the maltreatment group compared to the TD group (*U* = 95.50, *Z* = 1.72, *p* = .086). There were no significant differences between the autism and TD groups (*U* = 116.50, *Z* = 0.64, *p* = .524), or the autism and maltreatment groups (*U* = 44.50, *Z* = 0.75, *p* = .453).

***Removing Adolescents Meeting Clinical Cut-off in RCADS-11 (Youth Self-reports)***

The remaining participants per group were autism (*n* = 14), maltreatment (*n* = 12), and TD (*n* = 26). FT specificity scores were significantly lower in the maltreatment group compared to the TD group (*U* = 55.50, *Z* = 2.39, *p* = .017). There were no significant differences between the autism and TD groups (*U* = 132.00, *Z* = 0.04, *p* = .971), or the autism and maltreatment groups (*U* = 50.00, *Z* = 1.77, *p* = .077).

***Removing Adolescents Meeting Clinical Cut-off in CATS (Caregiver Reports)***

The remaining participants per group were autism (*n* = 22), maltreatment (*n* = 15), and TD (*n* = 28). FT specificity scores were significantly lower (one-tailed) in the maltreatment group compared to the TD group (*U* = 141.50, *Z* = 1.76, *p* = .079). There were no significant differences between the autism and TD groups (*U* = 233.50, *Z* = 1.47, *p* = .143), or the autism and maltreatment groups (*U* = 149.50, *Z* = 0.49, *p* = .627).

***Removing Adolescents Meeting Clinical Cut-off in CRIES (Youth Self-reports)***

The remaining participants per group were autism (*n* = 12), maltreatment (*n* = 13), and TD (*n* = 19). FT specificity scores were significantly lower in the maltreatment group compared to the TD group (*U* = 37.50, *Z* = 2.39, *p* < .001), and compared to the autism group (*U* = 32.50, *Z* = 2.51, *p* = .012). There were no significant differences between the autism and TD groups (*U* = 112.50, *Z* = 0.06, *p* = .951).

***Removing Adolescents with “Inconsistent” SCQ scores***

This involved removing those with SCQ scores below the cut-off in the autism group and above the cut-off in the maltreatment group. The remaining participants per group were autism (*n* = 20), maltreatment (*n* = 21), and TD (*n* = 28). FT specificity scores were significantly lower in the maltreatment group compared to the TD group (*U* = 134.50, *Z* = 3.75, *p* = .001). There were no significant differences between the autism and TD groups (*U* = 199.00, *Z* = 1.71, *p* = .088), or the autism and maltreatment groups (*U* = 160.50, *Z* = 1.31, *p* = .190).

***Removing Adolescents with “Low Motivation” (who did not Generate any Specific Event)***

The remaining participants per group were autism (*n* = 22), maltreatment (*n* = 20), and TD (*n* = 27). FT specificity scores were significantly lower in the maltreatment group compared to the TD group (*U* = 175.50, *Z* = 2.06, *p* = .040). There were no significant differences between the autism and TD groups (*U* = 112.50, *Z* = 0.06, *p* = .951), or the autism and maltreatment groups (*U* = 165.50, *Z* = 1.39, *p* = .165).

***Removing Maltreatment-Exposed Adolescents with Only Experiences of Neglect***

We excluded participants in the maltreatment group who experienced primarily neglect rather than abuse (Sheridan & McLaughlin, 2014), as previous work has indicated that reduced specificity may be specific to abused children (Valentino et al., 2006). The remaining maltreatment group was *n* = 16. FT specificity scores were significantly lower in the maltreatment group compared to the TD group (*U* = 120.00, *Z* = 2.56, *p* = .010). There were no significant differences between the maltreatment and autism groups (*U* = 202.50, *Z* = 0.71, *p* = .479).

***Removing Maltreatment-Exposed Adolescents Recruited Outside of Social Services***

As one may argue that maltreatment cases are not sufficiently “severe” unless seen within social services, we excluded participants in the maltreatment group who were *not* recruited from social services. The remaining maltreatment group was *n* = 23. FT specificity scores were significantly lower in the maltreatment group compared to the TD group (*U* = 168.50, *Z* = 2.93, *p* = .003). There were no significant differences between the maltreatment and autism groups (*U* = 276.00, *Z* = 1.07, *p* = .283).

***Comparing Number of Omissions***

The number of omissions (non-memories, i.e., “I can’t think of any event”; and accounting for SES and GCA) were not significantly different among the three groups, *H* = 5.87, *df* = 2, *p* = .053 (autism: *Mdn* = 2.00, *IQR* = 1.00-4.00; maltreatment: *Mdn* = 4.00, *IQR* = 2.00-6.00; TD: *Mdn* = 2.00, *IQR* = 1.00-4.00).

**Additional Results – Exploratory Analyses**

We repeated these using the number of overgeneral events (overgenerality scores) instead. In the maltreatment and autism groups, overgenerality scores were not significantly correlated with scores on the CRIES avoidance subscale, *r*(28) = 0.46, *p* = .013, or on the intrusion subscale, *r*’s = 0.12 to 0.34, *p*’s > .079. In the TD group, overgenerality scores were also not significantly correlated with avoidance subscale, *r*(28) = 0.35, *p* = .067, but significantly and positively correlated with intrusion subscale, *r*(28) = 0.46, *p* = .013, suggesting that a more overgeneral FT style was associated with more intrusions. This finding mirrors the pattern found with reduced specificity associated also with more intrusions.

Overgenerality scores were not significantly associated with executive function (DEX-C) in any group, *r*’s = -0.02 to 0.03, *p*’s > .830.

For mental health correlates, overgenerality scores were significantly and positively correlated with more symptoms of anxiety, *r*(85) = 0.22, *p* = .042, but not depression, *r*(83) = 0.15, *p* = .165, but the correlation with anxiety was rendered non-significant when accounting for SES and GCA, *r*(85) = 0.22, *p* = .061.

**References for Supplemental Materials**

Berument, S. K., Rutter, M., Lord, C., Pickles, A., & Bailey, A. (1999). Autism screening questionnaire: Diagnostic validity. *British Journal of Psychiatry*, *175*, 444–451. https://doi.org/10.1192/BJP.175.5.444

Chandler, S., Charman, T., Baird, G., Simonoff, E., Loucas, T., Meldrum, D., … Pickles, A. (2007). Validation of the Social Communication Questionnaire in a population cohort of children with autism spectrum disorders. *Journal of the American Academy of Child & Adolescent Psychiatry*, *46*(10), 1324–1332. https://doi.org/10.1097/CHI.0B013E31812F7D8D

Chesnut, S. R., Wei, T., Barnard-Brak, L., & Richman, D. M. (2017). A meta-analysis of the Social Communication Questionnaire: Screening for autism spectrum disorder. *Autism*, *21*(8), 920–928. https://doi.org/10.1177/1362361316660065

Chorpita, B. F., Moffitt, C. E., & Gray, J. (2005). Psychometric properties of the Revised Child Anxiety and Depression Scale in a clinical sample. *Behaviour Research and Therapy*, *43*(3), 309–322. https://doi.org/10.1016/j.brat.2004.02.004

Chorpita, B. F., Yim, L., Moffitt, C., Umemoto, L. A., & Francis, S. E. (2000). Assessment of symptoms of DSM-IV anxiety and depression in children: A revised child anxiety and depression scale. *Behaviour Research and Therapy*, *38*(8), 835–855. https://doi.org/10.1016/S0005-7967(99)00130-8

Deeba, F., Rapee, R. M., & Prvan, T. (2014). Psychometric properties of the Children’s Revised Impact of Events Scale (CRIES) with Bangladeshi children and adolescents. *PeerJ*, *2014*(1), e536. https://doi.org/10.7717/PEERJ.536/SUPP-2

Emslie, H., Wilson, F. C., Burden, V., Nimmo-Smith, I., & Wilson, B. A. (2003). *Behavioural Assessment of the Dysexecutive Syndrome for Children (BADS-C)*. Bury St. Edmunds: Thames Valley Test Company.

Esbjørn, B. H., Sømhovd, M. J., Turnstedt, C., & Reinholdt-Dunne, M. L. (2012). Assessing the revised child anxiety and depression scale (RCADS) in a national sample of Danish youth aged 8-16 years. *PLoS ONE*, *7*(5). https://doi.org/10.1371/journal.pone.0037339

Giannopoulou, I., Smith, P., Ecker, C., Strouthos, M., Dikaiakou, A., & Yule, W. (2006). Factor structure of the Children’s Revised Impact of Event Scale (CRIES) with children exposed to earthquake. *Personality and Individual Differences*, *40*(5), 1027–1037. https://doi.org/10.1016/J.PAID.2005.11.002

Lord, C., Rutter, M., & Le Couteur, A. (1994). Autism Diagnostic Interview-Revised: A revised version of a diagnostic interview for caregivers of individuals with possible pervasive developmental disorders. *Journal of Autism and Developmental Disorders*, *24*(5), 659–685. https://doi.org/10.1007/BF02172145

Nilsson, D., Dävelid, I., Ledin, S., Carl, &, Svedin, G., D€, I., … Svedin, O. (2020). Psychometric properties of the Child and Adolescent Trauma Screen (CATS) in a sample of Swedish children. *Nordic Journal of Psychiatry*, *75*(4), 247–256. https://doi.org/10.1080/08039488.2020.1840628

Perrin, S., Meiser-Stedman, R., & Smith, P. (2005). The Children’s Revised Impact of Event Scale (CRIES): Validity as a screening instrument for PTSD. *Behavioural and Cognitive Psychotherapy*, *33*(4), 487–498. https://doi.org/10.1017/S1352465805002419

Radez, J., Waite, P., Chorpita, B., Creswell, C., Orchard, F., Percy, R., … Reardon, T. (2021). Using the 11-item version of the RCADS to identify anxiety and depressive disorders in adolescents. *Research on Child and Adolescent Psychopathology*, *49*(9), 57. https://doi.org/10.1007/S10802-021-00817-W

Rutter, M., Bailey, A., & Lord, C. (2003). *The Social Communication Questionnaire*. Los Angeles, CA: Western Psychological Services.

Sachser, C., Berliner, L., Holt, T., Jensen, T. K., Jungbluth, N., Risch, E., … Goldbeck, L. (2017). International development and psychometric properties of the Child and Adolescent Trauma Screen (CATS). *Journal of Affective Disorders*, *210*, 189–195. https://doi.org/10.1016/j.jad.2016.12.040

Sheridan, M. A., & McLaughlin, K. A. (2014). Dimensions of early experience and neural development: Deprivation and threat. *Trends in Cognitive Sciences*, *18*(11), 580–585. https://doi.org/10.1016/J.TICS.2014.09.001

Verlinden, E., van Meijel, E. P. M., Opmeer, B. C., Beer, R., de Roos, C., Bicanic, I. A. E., … Lindauer, R. J. L. (2014). Characteristics of the Children’s Revised Impact of Event Scale in a clinically referred Dutch sample. *Journal of Traumatic Stress*, *27*(3), 338–344. https://doi.org/10.1002/JTS.21910
